# Recruitment in randomized clinical trials: The MeMeMe experience

**DOI:** 10.1371/journal.pone.0265495

**Published:** 2022-03-25

**Authors:** Ivan Baldassari, Andreina Oliverio, Vittorio Krogh, Eleonora Bruno, Giuliana Gargano, Mauro Cortellini, Alice Casagrande, Maria G. Di Mauro, Elisabetta Venturelli, Daniela Del Sette Cerulli, Bellegotti Manuela, Franco Berrino, Patrizia Pasanisi

**Affiliations:** Department of Research, Epidemiology and Prevention Unit, Fondazione IRCCS Istituto Nazionale dei Tumori di Milano, Milan (MI), Italy; Universidade Federal de Goias, BRAZIL

## Abstract

**Introduction:**

Recruitment is essential for the success of clinical trials. We are conducting a randomized clinical trial to test the effect of a Mediterranean dietary intervention with or without 1700 mg/day of metformin for the prevention of age-related chronic diseases, the MeMeMe trial (Trial registration number: EudraCT number: 2012-005427-32 ClinicalTrials.gov ID: NCT02960711). MeMeMe recruiting experience, highlighting strengths, limitations encountered and results is reported.

**Patients and methods:**

Statistical analysis focused on the reasons for withdrawal according to the recruitment method (“active” *versus* “passive” criterion) and the time of withdrawal. Logistic regression models were used to explore the associations between the risk of withdrawal and sex, recruitment method, randomization arm, and with markers of compliance to the intervention, such as one-year change in body weight.

**Results:**

Out of 2035 volunteers, 660 (32.4%) were recruited “actively” and 1375 (67.6%) “passively”. Among people who dropped out of the trial after randomization, there were 19.5% for the “active” and 22.0% for the “passive” method (p = 0.28). The risk of withdrawal was significantly higher in women (OR:1.91; 95% CI:1.17–3.12; p = 0.01), in volunteers older at recruitment (OR:1.25; 95% CI:1.07–1.45; p = 0.004), and in those with a higher BMI at baseline (OR:1.23; 95% CI:1.07–1.43; p = 0.004). Volunteers who lost at least 2 kg (the median weight change) in the first year of intervention were significantly less (53%) likely to withdraw from the trial (OR:0.48; 95% CI:0.30–0.75; p = 0.001).

**Conclusion:**

Our findings suggest that the “passive” recruitment method was more effective than the “active” one to advance recruitment. The benefits of “passive” recruitment hardly outweighed the drawbacks.

**Trial registration:**

**Trial registration number**: EudraCT number: 2012-005427-32. ClinicalTrials.gov ID: NCT02960711.

## Introduction

Randomized controlled trials (RCTs) are widely accepted as the most powerful research method for minimizing bias when evaluating treatments, technologies, and programs for health. High-quality RCTs have to include appropriate randomization (balancing confounders in the randomized groups), blinding, and full follow-up of all patients for outcome assessment; for the intervention arms, high intervention rates are important to maximize the ability to assess the effect of the intervention. [1]. However, ethics, recruitment hitches, sampling bias, patient resistance and treatment comparisons may limit the design and conduction in practice of RCTs [2].

Among RCTs, clinical trials (CTs) for the primary prevention of diseases require large sample sizes (with high enrolment rates) and long follow-up to ensure high- quality results. Recruitment methods are therefore a critical topic for the success of CTs. Poor timing and insufficient recruitment have negative impacts on the costs of CTs, on workload [3,4] and on scientific quality [2,5]. In fact, although proper randomization may protect internal validity in trials with low enrolment rates, high enrolment rates improve the generalizability of the intervention and the results [6].

We conducted a RCT in Italy to test the effect of a Mediterranean dietary intervention (MedDiet) with or without 1700 mg/day of metformin (MET) on the incidence and mortality for age-related chronic diseases in volunteers at high risk because of age (50–79 at the start of the study) and the presence of metabolic syndrome (MetS): the MeMeMe trial (NCT02960711), standing for MetS, MedDiet and MET [7–9].

MeMeMe required a substantial management effort, involving extensive organizational, economic and informatic investments. Recruitment, the sequential administration of drug/placebo [10], the management of drug supply and participants staying in the trial, were the main critical points. The present paper describes the MeMeMe recruiting experience, highlighting strengths, limitations encountered, and results.

## Material and methods

Detailed information about the MeMeMe design and the study protocol have already been described. [7–9] Briefly, MeMeMe is a phase III randomized controlled trial whose design is 2x2 factorial with 1600 volunteers (according to amendment no.1 to grant agreement 322752) to be randomized in four groups of 400 each and allocated to the following treatments:

- MET (1700 mg/day) + active Mediterranean dietary intervention
- Placebo + active Mediterranean dietary intervention
- MET (1700 mg/day) alone
- Placebo alone.

The MET/placebo component is double-blind.

In accordance with the inclusion criteria of the trial, volunteers were men and women aged 50–79 with a MetS. Subjects were excluded for diabetes, renal, cardiac or hepatic insufficiency, cancer diagnosed in the last five years, concomitant treatment with potassium-sparing diuretics or proton pump inhibitors, and excessive alcohol consumption. Anyone with distressing side effects of MET during the first 30 days of 500 mg/day of MET was not included.

The volunteers were fully informed about the trial and gave signed informed consent. They were required:

- to complete a personal data form;
- to complete a form including medical history, behavioral factors and reasons for entry to the trial. Participants could indicate more than one reason for entry;
- to attend a clinical visit for anthropometric and body composition measurements;
- to provide a 20-mL blood sample for markers of MetS and hormonal assays (plasma glucose, triglycerides, total, LDL and HDL cholesterol and serum insulin);
- to provide information on their health status, and to permit the trial investigators to contact their general practitioners, consult clinical notes and examine biopsy material, as necessary;
- to complete a 24-hour food frequency diary of the previous day—the validated 14-point MEDAS questionnaire [11], the validated Pittsburgh Sleep Quality questionnaire (PSQI) [12], the reduced morning-evening questionnaire (r-MEQ) [13] and the Godin Leisure-Time Exercise Questionnaire [14].

At recruitment all participants received general recommendations for lifestyle prevention of cancer. [15]. After the baseline screening examinations, participants were tested for low-dose MET tolerance (MET 500 mg/day for 30 days) and were then randomized to either the placebo group or the MET group. After randomization, participants were invited to take one tablet of MET/placebo (850 mg/day) for another two months and then to start the full treatment (two tablets for a total of 1700 mg/day MET).

Both groups were also randomized to the active MedDiet or to a control group that continued applying the baseline recommendations. Volunteers randomized to the MedDiet group were invited to participate in dietary activities with common lunch/dinner and kitchen classes at least once a month.

Anthropometric measurements, blood samples and dietary data were collected at baseline, after one year and at the end of follow-up.

MeMeMe was supported by an ERC grant and approved by the institutional review board and ethical committee of the Fondazione IRCCS Istituto Nazionale dei Tumori di Milano (approval number: INT 85/13). The trial started in August 2013 and was planned to last five years, including three years of recruitment. However, due to problems with authorization for the drug and an initial 12-month delay in drug supply, the scheduled period was extended (including recruitment) and the MeMeMe was formally concluded on 31 July 2019 (amendment no.1 to grant agreement 322752). The participants’ follow-up is now being completed (till 31 December 2021).

### Recruitment methods

The MeMeMe is a monocentric trial with a single recruitment center at the Fondazione IRCCS Istituto Nazionale dei Tumori di Milano. Recruitment was conducted from 1 May 2014 to 30 June 2016 following a selective “active” criterion, involving general practitioners, blood donation centers in Lombardy and clinical units dealing with overweight and metabolic diseases. People interested were contacted and invited to attend meetings at which the researchers illustrated the trial design and protocol and briefly checked the applicants’ waist circumference using a paper tape.

New recruitment strategies were adopted from 1 July 2016 to 30 June 2018, following a “passive” criterion, mainly involving media activities. We started to use social networks, put posters on billboards at the Milan underground stations and in shopping centers, and advertised the trial in the main local and national newspapers. PI participated in interviews on television and local/national radio and researchers organized open days, demonstrating what would be done in the trial. All these activities passively awaited contact from people interested.

### Statistical analysis

The analyses included all volunteers who completed the personal data and medical history forms. The personal characteristics of the MeMeMe population were reported as frequencies. The metabolic and anthropometric characteristics were summarized by reason for entry, using means and standard deviation (SD) or frequencies, and compared using *t* tests or χ^2^, as appropriate. Body mass index (BMI) was defined as body weight/height squared (kg/m^2^). All the variables showed normal distribution, with no need for transformation. Normality was firstly graphically checked by using histograms. Furthermore, we also used the Shapiro-Wilk test to confirm the normality of the parameters under study.

Statistical analysis focused on the reasons for withdrawal from the trial according to the recruitment method (people recruited through an “active” criterion compared with those recruited “passively”) and the time of withdrawal. We summarized four potential withdrawal times:

- T0: before the baseline screening examinations
- T1: after the baseline screening examinations but before randomization to MET/placebo
- T2: before the one-year examinations
- T3: after the one-year examinations

Logistic regression models were used to explore the associations between the risk of withdrawal from the trial and sex, recruitment method, randomization arm, and with markers of compliance to the dietary intervention, such as one-year change in body weight. Age (quintiles) and BMI at baseline (quintiles) were retained as covariates in the models. All the assumptions underlying the logistic regression model were firstly graphically checked and verified.

A p-value <0.05 was taken as significant. All statistical tests were two-sided. Analyses were done using the STATA 16 statistical package.

## Results

**Fig 1:**
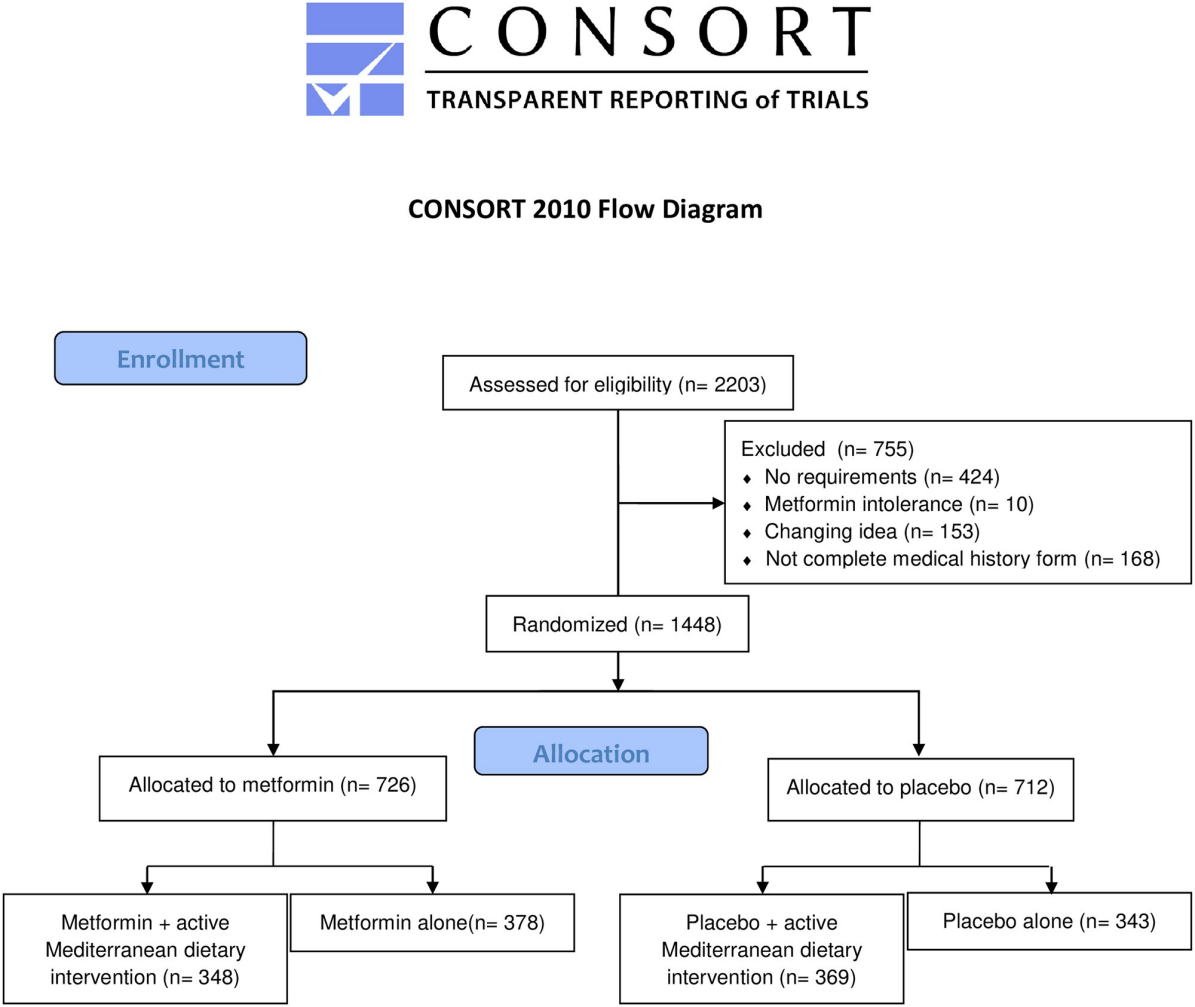
Flowchart of the MeMeMe study.

Out of the 2203 potentially eligible volunteers who gave signed informed consent and supplied their personal data, 2035 completed the medical history form and 1994 participated in the baseline screening, blood sampling, anthropometric and clinical examinations (Fig 1).

After the baseline examinations and the 30 days test with MET 500 mg/day, 1448 people were properly randomized (Fig 1).

The present analysis refers to the 2035 people who completed the personal data and medical history forms. Among these participants, there were 64.6% women (mean age 62.2 ± 6.8 years) and 35.4% men (mean age 63.4 ± 6.7 years); 47.2% of the study population was in the age class 60–69 years. Most of the people were married and had a high school diploma. On the whole, adhering to a “healthy lifestyle” was the most frequent reason given for entry (Table 1).

**Table 1:** General characteristics of the MeMeMe population.

		No.	%
**Age (years)**	50–59	719	35.3
	60–69	960	47.2
	70–80	356	17.5
**Sex**	Male	721	35.4
	Female	1314	64.6
**Marital Status**	Unmarried	337	16.6
	Married	1366	67.3
	Divorced	216	10.7
	Widow/er	110	5.4
**Education**	Low	344	16.5
	High	1061	53.0
	Degree or more	607	30.5
**Profession**	Employee	763	37.7
	Freelance	272	13.4
	Retired	809	40.0
	Other	180	8.9
**Concern about health**	YES	807	39.7
	NO	1224	60.3
**Help research**	YES	723	35.6
	NO	1308	64.4
**Healthy lifestyle**	YES	1642	80.8
	NO	389	19.2

**Note**: Medical history was not complete for all participants.

Among the volunteers who selected as response in the personal data form one single reason for entering the trial, 811 (73.5%) indicated “healthy lifestyle”, 215 (19.5%) “concern about health” and 78 (7.0%) “help research” (data not in table).

Table 2 reports the anthropometric and metabolic characteristics of the whole MeMeMe population by reason for entry. Participants who entered the trial ticking “concern about health” had significantly higher weight (p<0.001), BMI (p<0.001), waist and hip circumferences (both p<0.001) than people who did not give this reason. As regards the metabolic parameters too, they had significantly higher levels of glucose (p = 0.02) and triglycerides (p = 0.03), with a borderline significantly larger number of MetS factors (p = 0.06) compared to people who did not tick “concern about health”.

**Table 2:** Metabolic characteristics of the MeMeMe population by reason for entry.

	Concern about health^+^		Help Research^+^		Healthy Lifestyle^+^	
	Yes (792)	No (1197)	Yes (704)	No (1285)	Yes (1607)	No (382)
**Weight (kg)**	83.6 ± 17.2*	81.3 ± 15.4*	82.0 ± 15.5	82.4 ± 16.5	82.3 ± 15.9	82.0 ± 17.2
**BMI**	31.3 ± 5.7*	30.0 ± 4.5*	30.4 ± 5.0	30.6 ± 5.1	30.4 ± 5.0	30.9 ± 5.6
**Waist circumference (cm)**	98.6 ± 13.6*	96.7 ± 12.1*	97.0 ± 12.2	97.7 ± 13.0	97.6 ± 12.6	97.0 ± 13.1
**Hip circumference (cm)**	107.6 ± 11.5*	105.3 ± 8.9*	105.9 ± 9.7	106.4 ± 10.4	106.1 ± 9.6	106.9 ± 12.1
**Systolic blood pressure (mmHg)**	145.9 ± 19.4	145.3 ± 19.4	144.9 ± 18.9	145.9 ± 19.7	145.7 ± 19.3	144.9 ± 20.1
**Diastolic blood pressure (mmHg)**	87.7 ± 10.4	87.0 ± 9.8	87.2 ± 10.1	87.3 ± 10.0	87.4 ± 10.0	86.9 ± 10.3
**Glycemia (mg/dL)**	101.5 ± 16.2*	100.0 ± 13.4*	100.3 ± 12.6	100.7 ± 15.6	100.6 ± 14.0	100.6 ± 16.7
**Total cholesterol (mg/dL)**	209.1 ± 36.3	211.9 ± 35.9	210.5 ± 35.9	210.9 ± 36.1	211.3 ± 36.2	208.6 ± 35.3
**HDL cholesterol (mg/dL)**	57.2 ± 14.8	57.5 ± 14.7	57.7 ± 14.5	57.2 ± 14.9	57.3 ± 14.9	58.0 ± 14.1
**LDL cholesterol (mg/dL)**	127.5 ± 32.0*	131.1 ± 32.0*	129.4 ± 31.4	129.8 ± 32.4	130.3 ± 32.2	126.9 ± 31.2
**Triglycerides (mg/dL)**	121.4 ± 61.8*	115.8 ± 54.3*	116.6 ± 55.5	118.8 ± 58.5	118.4 ± 58.9	116.4 ± 50.9
**Creatinine (mg/dL)**	0.8 ± 0.2	0.8 ± 0.2	0.8 ± 0.2	0.8 ± 0.2	0.8 ± 0.2	0.8 ± 0.2
**AST (U/L)**	20.3 ± 6.0	20.5 ± 6.3	20.3 ± 5.7	20.4 ± 6.5	20.4 ± 6.2	20.3 ± 6.6
**ALT (U/L)**	23.1 ± 12.8	22.5 ± 11.6	22.5 ± 11.2	22.9 ± 12.5	22.6 ± 11.5	22.6 ± 14.3
**γGT (U/L)**	31.3 ± 26.6	30.0 ± 24.3	29.2 ± 20.9	31.3 ± 27.3	30.7 ± 25.3	29.7 ± 24.8
**Hypercholesterolemia therapy (%)**	26.6	23.1	24.6	24.4	23.9	27.0
**Hypertension therapy (%)**	52.8	49.1	49.3	51.3	50.6	50.5
**Hypertriglyceridemia therapy (%)**	3.3	2.5	2.8	2.8	2.7	3.4
**MetS (%)**						
0	0.4	0.2	0.0	0.4	0.2	0.3
1–2	17.6	21.7	21.5	19.3	20.2	19.6
3–5	82.0	78.1	78.5	80.3	79.6	80.1

**Notes**:

^+^ data available for the 1989 volunteers who indicated their reasons for entry.

* statistically significant.

**Fig 2:**
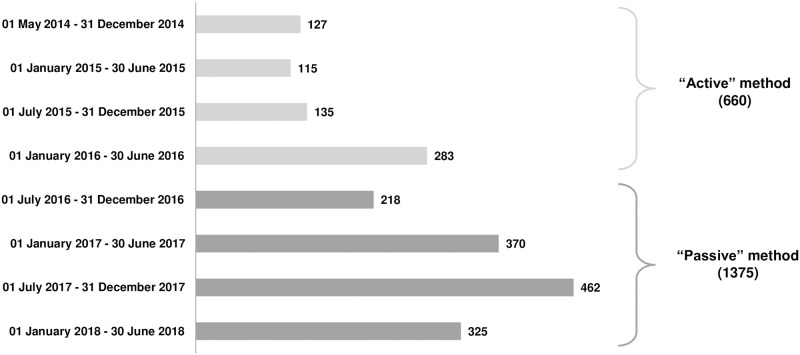
Distribution of volunteers (by semester) over the four-year of recruitment period.

Among the 2035 participants, 660 volunteers (32.4%) were recruited “actively” (i.e. directly contacting volunteers through clinical centers and general practitioners) and 1375 (67.6%) “passively” (i.e. mainly involving media activities). Fig 2 reports by semester the distribution of volunteers over the four-year of recruitment period (from May 2014 to June 2018) according to the “active” (before June 2016) *versus* the “passive” (after June 2016) method of recruitment.

Table 3 reports the distribution of individuals who left the trial, according to reason and time of withdrawal (T0, T1, T2 and T3), by recruitment method (“active” or “passive”).

**Table 3:** Distribution of withdrawals according to reason and time, by recruitment method.

Reason (%)	T0 (41)		T1 (546)		T2 (187)		T3 (118)	
	Active (24)	Passive (17)	Active (135)	Passive (411)	Active (52)	Passive (135)	Active (45)	Passive (73)
**Changing idea (Not specified)**	100.0*(24)	64.8* (11)	24.4 (33)	14.8 (61)	63.5 (33)	74.1 (100)	73.3 (33)	64.4 (47)
**Changing idea (Family/job)**	0 (0)	17.6 (3)	3.0 (4)	2.2 (9)	17.3 (9)	14.1 (19)	24.5 (11)	21.9 (16)
**Changing idea (Medical advice)**	0 (0)	17.6 (3)	0.8 (1)	1.2 (5)	1.9 (1)	2.9 (4)	0.0 (N0)	2.7 (2)
**No MetS**	ND	ND	69.6* (94)	80.1* (329)	ND	ND	ND	ND
**MET Intolerance**	ND	ND	2.2 (3)	1.7 (7)	17.3 (9)	8.8 (12)	2.2 (1)	11.0 (8)

**Note**:

* statistically significant.

At T0 41 individuals left the trial, refusing the baseline screening examinations. At T0 all the volunteers recruited through the “active” method left the trial for “changing idea” without specifying their reason. At T1 546 volunteers left the trial, before randomization, mainly due to a lack of inclusion criteria: people recruited through the “passive” method did not have MetS significantly more than people recruited “actively” (p<0.001). We did not find any other significant difference between the two groups.

At T2 and T3 305 volunteers left the trial after randomization, 68% of whom had been recruited through the “passive” method. Among the 1448 subjects randomized, 497 were recruited through the “active” method and 951 through the “passive” approach. Therefore, as regards people who actually dropped out of the trial after randomization (at T2 or T3), there were 19.5% for the “active” and 22.0% for the “passive” methods (p = 0.28).

Among these 1448 participants randomized, 30 (2%) left the trial for intolerance to MET (27 in the MET group and 3 in the placebo group), and 275 (19.0%) for “changing idea”. About half the participants who withdrew from the trial for “changing idea” were in the placebo group (51%) and half (49%) in the MET group.

We used a logistic regression model to explore in the whole population the associations between the risk of withdrawal from the trial after randomization, and sex, recruitment method, randomization arm and a marker of compliance to the intervention, such as the one-year change in body weight. The risk of withdrawal was significantly higher in women (OR:1.91; 95% CI:1.17–3.12; p = 0.01), in volunteers who were older at recruitment (OR:1.25; 95% CI:1.07–1.45; p = 0.004), and in those with a higher BMI at baseline (OR:1.23; 95% CI:1.07–1.43; p = 0.004). We also noted that volunteers who lost at least 2 kg (the median weight change) in the first year of intervention were significantly less (53%) likely to withdraw from the trial (OR:0.48; 95% CI:0.30–0.75; p = 0.001).

Belonging to the group recruited through the “passive” method was not significantly associated with a lower OR of withdrawal (OR:0.77; 95% CI:0.51–1.18; p = 0.23). We found no association with randomization arm.

## Discussion

Our findings suggest that people who adhered to our trial of MedDiet and MET interventions entered mainly because of interest in changing their lifestyle. In line with this, volunteers who left the trial after randomization were more frequently participants who failed to lose weight after one year of intervention.

The decision to start, continue, or withdraw from a trial in a population setting is often based on multiple factors, with significant variability across studies. In the MeMeMe, a higher BMI at baseline and older age at recruitment were significantly associated with higher risks of withdrawal.

Our findings also suggest that volunteers who decide to participate in a prevention trial tend to drop out when they do not see any early beneficial effect. In fact, although the goal of the MeMeMe is the prevention of chronic diseases over time, the lack of weight loss at the end of the first year of intervention was an important risk factor for withdrawing from the study, especially for women. This should teach us to be even more careful in explaining to volunteers the aims of the trial, the importance of remaining in it despite their perceived results, and in understanding each participant’s actual motivations.

The results also suggest that the “passive” recruitment method was more effective than the “active” one to boost the numbers of recruits. However, among people who did not enter randomization (T1) because they did not meet the MetS criteria, 75% had been recruited “passively”.

This picture outlines a complex scenario for RCT researchers. On one hand, the active search and recruitment of targeted candidates, with the help of general practitioners and clinical units dealing with overweight and metabolic diseases, might be useful for smooth admission of participants into the trial, but this approach risks giving a very low enrollment rate. On the other hand, passive recruitment using advertising in newspapers and mass media, with less concern about their metabolic condition and/or their motivation for entering the trial, might give a better enrollment rate, but it may result in fewer participants being randomized because of the lack of inclusion criteria or higher drop-out rate, or both.

Despite these considerations and in line with the recruitment methods of other health programs [16,17], in our trial the benefits of “passive” recruitment hardly outweighed the drawbacks. In fact, considering people who actually left the trial after randomization (T2 and T3), the frequency by recruitment method did not substantially differ (19.5% for “active” and 22.0% for “passive”).

A previous study suggested that the use of monetary incentives helps investigators in recruiting [18] but this practice is unusual in Italy and we did not provide any money for the participants. As for the costs of “passive” recruitment (advertising, billboards, posters, etc.), we spent less than 2% of the total ERC funding. If we had allocated 5% of the total funding (100,000 Euro) exclusively for mass media advertising, we would have reached the recruitment objective in a shorter time.

After randomization, 87% of participants attended the one-year MeMeMe examinations, a smaller proportion than in another big primary prevention lifestyle trial such as the PREDIMED [19] but quite similar to a larger trial with a mixed intervention (MET and lifestyle) for the primary prevention of type-2 diabetes [20]. In all, 21% of volunteers left the study after randomization (19% for “changing idea” and 2% for MET intolerance) and this was lower than in the Diabetes Prevention Program.

Gastrointestinal adverse events are typical of MET treatment, in particular diarrhea, nausea, and abdominal discomfort. Trials in diabetics reported a prevalence of 15–20% of these adverse events in patients given MET 1500 mg/day or more. In our trial, only 2% of people taking MET 1700 mg/day withdrew. Probably the 30 days of MET 500 mg/day (trial period) served to exclude any intolerant subjects before randomization, and increasing the dosage of MET/placebo (one 850 mg tablet/day of MET/placebo) for the first two months after randomization, before starting the full treatment, helped reduce the prevalence of gastrointestinal adverse events.

Our findings about recruitment and withdrawal might have low external validity, given the setting and population focus, and this is the limit of the present analysis. However, we think that our experience might be useful to researchers who involve in clinical trials healthy subjects. In fact, our results suggest that healthy people are probably more attracted to messages from mass media/social channels rather than communications from healthcare professionals.

MeMeMe is a unique, innovative trial that includes two types of intervention, using MET with or without the MedDiet for primary prevention in people at risk because of age (>50 years) and the presence of a MetS. Therefore, people who volunteer to enter a trial that involves two almost opposing preventive interventions—the use of a drug or the change of dietary habits—have multiple reasons for entry and individual objectives.

## Conclusions

In the MeMeMe trial the “passive” recruitment method was more effective than the “active” one to advance recruitment and boost the number of volunteers. Furthermore, the benefits of “passive” recruitment hardly outweighed the drawbacks. People who adhered to the trial entered mainly because of interest in changing their lifestyle. In line with this, volunteers who left the trial after the randomization were more frequently participants who failed to lose weight after one year of intervention.

MeMeMe is currently completing the three years of follow-up (on average). We believe this trial will help clarify the importance of the MedDiet for primary prevention and the role of MET as a potential chemopreventive agent.

## Supporting information

S1 Checklist CONSORT 2010 checklist of information to include when reporting a randomised trial*. (DOC)

S1 File (PDF)
