# Supplementary material for: Recruitment in randomized clinical trials: The MeMeMe experience
Source: PLoS One. 2022 Mar 25;17(3):e0265495. doi: 10.1371/journal.pone.0265495 (PMC8956174; doi:10.1371/journal.pone.0265495)
Supplement: S1 File — (PDF) [file pone.0265495.s002.pdf]

SEVENTH FRAMEWORK PROGRAMME OF THE EUROPEAN UNION

EUROPEAN RESEARCH COUNCIL EXECUTIVE AGENCY

SP2-Ideas

Support for frontier research (ERC)

ERC Advanced Grant

ERC-2012-ADG_20120314

Grant Agreement Number 322752

MeMeMe

Randomized controlled trial of metformin and dietary restriction to prevent age-related morbid events in people with metabolic syndrome

SEVENTH FRAMEWORK PROGRAMME

ERC GRANT AGREEMENT No 322752

PROJECT TITLE MeMeMe

Support for frontier research (ERC)

ERC Advanced Grant

The **European Research Council Executive Agency** (the "*Agency*"), acting under powers delegated by the European Commission (the "*Commission*")

of the **one part**,

and FONDAZIONE IRCCS ISTITUTO NAZIONALE DEI TUMORI, established in Via Venezian 1, Milan, 20133, Italy represented by Giuseppe De Leo, President or his authorised representative, (the "*beneficiary*"),

of the **other part**

HAVE AGREED to the following terms and conditions including those in the following annexes, which form an integral part of this *grant agreement* (the "*grant agreement*").

Annex I - Description of Work
Annex II - ERC General Conditions - Single Beneficiary
Annex III - ERC accession forms for new and other *beneficiaries* to the *grant agreement*.
Annex IV - Financial statement form.
Annex V - a) 'Terms of reference for the certificate for the financial statements' and
b) 'Terms of reference for the certificate on the methodology'

Article 1 - Scope

The European Union ("*the Union*"), has decided to grant a financial contribution for the implementation of the *project* as specified in Annex I, called *Randomized controlled trial of metformin and dietary restriction to prevent age-related morbid events in people with metabolic syndrome* (*MeMeMe*) (the "*project*") within the framework of the *SP2-Ideas* and under the conditions laid down in this *grant agreement.*

Article 2 - The *principal investigator*

1. The "*principal investigator*" as defined in Annex II is Dr. FRANCO BERRINO, born in FORNOVO DI TARO (PR), Italy, on 30/04/1944.

2. The *beneficiary* shall enter into a *supplementary agreement* with the "*principal investigator*". The provisions of the *supplementary agreement*, which are not in accordance with this *grant agreement*, shall be deemed to be void for the purposes of this *grant agreement.*

3. Together with the signed *grant agreement* the *beneficiary* shall transmit to the *Agency* a signed copy of this *supplementary agreement.*

Article 3 - Duration and *start date* of the *project*

The duration of the *project* shall be 60 months from 1st August 2013 (hereinafter referred to as the "*start date*").

Article 4 - Reporting periods and language of reports

1. The *project* is divided into scientific reporting periods of the following duration:

- PA: from month 1 to month 30
- PB: from month 31 to the last month of the *project*.

Any scientific report required by this *grant agreement* shall be in English.

2. The *project* is divided into financial management reporting periods of the following duration:

- P1: from month 1 to month 18
- P2: from month 19 to month 36
- P3: from month 37 to month 54
- P4: from month 55 to the last month of the *project*.

Any financial management report required by this *grant agreement* shall be in English.

Article 5 - Maximum financial contribution of *the Union*

1. The maximum financial contribution of *the Union* to the *project* shall be EUR 2,499,999.60 (*two million four hundred and ninety nine thousand nine hundred and ninety nine EURO and sixty cents*). The actual financial contribution of *the Union* shall be calculated in accordance with the provisions of this *grant agreement*.

2. The financial contribution of *the Union* shall be in the form of a grant to the budget as specified in the table indicating the estimated breakdown of budget included in Annex I.

3. The bank account of the *beneficiary* to which all payments of the financial contribution of *the Union* shall be made is:

Name of account holder: Fondazione IRCCS "Istituto Nazionale Tumori"
Name of bank: Banca Popolare di Sondrio
Account reference: IT15C0569601620000002001X82

Article 6 - Pre-financing

1. A pre-financing of EUR 1,000,000.00 (*one million EURO*) shall be paid to the *beneficiary* within 30 days following the date of entry into force of this *grant agreement*.

2. The *beneficiary* hereby agrees that the amount of EUR *124,999.98* (*one hundred and twenty four thousand nine hundred and ninety nine EURO and ninety eight cents*), corresponding to the *beneficiary's* contribution to the Guarantee Fund referred to in Article II.20 and representing 5% of the maximum financial contribution of *the Union* referred to in Article 5.1, is transferred in their name by the *Agency* from the pre-financing into the Guarantee Fund. However, the *beneficiary* is deemed to have received the full pre-financing referred to in the first indent and will have to justify it in accordance with this *grant agreement.*

Article 7 - Special clauses

The following special clauses apply to this *grant agreement*:

Special clause 6

Notwithstanding the provisions of Article 6 the pre-financing shall be paid not earlier than 45 days before the *start date* of the *project.*

Special clause 15 ERC

1. The *beneficiary(ies)* shall provide the *Agency* with a written confirmation that it has received (a) favourable opinion(s) of the relevant ethics committee(s) and, if applicable, the regulatory approval(s) of the competent national or local authority(ies) in the country in which the research is to be carried out before beginning any *Agency* approved research requiring such opinions or approvals. The copy of the official approval from the relevant national or local ethics committees must also be provided to the *Agency.*

Special clause 39 ERC

In addition to Article II.30.3, the *beneficiary* shall deposit an electronic copy of the published version or the final manuscript accepted for publication of a scientific publication relating to *foreground* published before or after the final report in an institutional or subject-based repository at the moment of publication.

The *beneficiary* is required to make its best effort to ensure that this electronic copy becomes freely and electronically available to anyone through this repository:

- immediately if the scientific publication is published "open access", i.e. if an electronic version is also available free of charge via the publisher, or
- within 6 months of publication.

Article 8 - Communication

1. Any communication or request concerning the *grant agreement* shall identify the *grant agreement* number, the nature and details of the request or communication and be submitted to the following addresses:

For the *Agency*: European Commission
European Research Council Executive Agency
ERC Executive Agency - C2, COV2
Rue de la Loi 200
B-1049 Brussels, Belgium

For the *beneficiary*: Dr. Antonio Cannarozzo
FONDAZIONE IRCCS ISTITUTO NAZIONALE DEI TUMORI
Direzione Amministrativa / s.c. Affari Generali e Legali
Via Venezian 1
Milano 20133
ITALY

2. Reports and deliverables shall be transmitted to the *Agency* according to Article II.4.6.

3. For information or documents to be transferred by e-mail, the following addresses shall be used:

For the *Agency*: ERC-C2@ec.europa.eu

For the *beneficiary*: antonio.cannarozzo@istitutotumori.mi.it

4. In case of refusal of the notification or absence of the recipient, the *beneficiary* is deemed to have been notified on the date of the latest delivery, if notification has been sent to one of the addresses mentioned in paragraphs 1 and 3 and to its legal representative.

5. Any communication or request relating to the processing of personal data (Article II.13) shall be submitted, using the address(es) for the *Agency* identified in paragraphs 1 and 3, to the controller responsible for the processing: Head of Department "Grant Management"

Article 9 - Applicable law and competent court

The financial contribution of *the Union* is a contribution from *the Union* research budget with the aim to implement the 7th Research Framework Programme (FP7) and it is incumbent on the *Agency* and the Commission to execute FP7. Accordingly, this *grant agreement* shall be governed by the terms of this *grant agreement*, the European Community and European Union acts related to FP7, the *Financial Regulation* applicable to the general budget of *the Union* and its *Rules of Application* and other European Community and European Union law and, on a subsidiary basis, by the law of Belgium.

Furthermore the *beneficiary* is aware and agrees that the *Commission* may take a decision to impose pecuniary obligations, which shall be enforceable in accordance with Article 299 of the Treaty on the Functioning of the European Union.

Notwithstanding the *Commission's* right to adopt directly recovery decisions referred to in the previous paragraph, the General Court, or on appeal, the Court of Justice of the European Union, shall have sole jurisdiction to hear any dispute between *the Union* and a *beneficiary* concerning the interpretation, application or validity of this *grant agreement* and the validity of the decision mentioned in the second paragraph

Article 10 - Application of the *grant agreement* provisions

Any provision of this part of the *grant agreement*, shall take precedence over the provisions of the Annexes and the provisions of Annex II shall take precedence over the provisions of Annex I.

The special clauses set out in Article 7 shall take precedence over any other provisions of this *grant agreement*.

Article 11 - Entry into force of the *grant agreement*

This *grant agreement* shall enter into force after its signature by the *beneficiary* and the *Agency*, on the day of the last signature.

Done in two originals in English.

SEVENTH FRAMEWORK PROGRAMME

"Ideas" Specific programme

European Research Council

Grant agreement for Advanced Grant

Annex I - "Description of Work"

Project acronym: MeMeMe
Project full title: Randomized controlled trial of metformin and dietary restriction to prevent age-related morbid events in people with metabolic syndrome
Grant agreement no.: 322752
Duration: 60 months

Date of preparation of Annex I (latest version): 15 May 2013

Principal Investigator: Franco Berrino, MD
Host Institution: Fondazione IRCCS Istituto Nazionale dei Tumori

TABLE OF CONTENT:

Curriculum Vitae .... pg 3

funding ID .... pg 4

10-Year-Track-Record .... pg 6

Background and Objectives .... pg 8

Methodology .... pg 11

Team .... pg 16

Operative Protocol .... pg 17

Randomization .... pg 18

Funding .... pg 19

Budget Tables .... pg 20

Ethics Issues Table........ pg 22

Curriculum Vitae

Franco Berrino (FB), Italian, born in 1944, MD (1969) PhD in pathology (1975), epidemiologist, has been active on cancer research and chronic disease prevention for almost 40 years, with over 350 peer-reviewed scientific papers.

In the 1970s FB established the **first Italian cancer registry in the Varese Province** (IARC *Cancer Incidence in Five Continents, Volumes V to IX*) on the basis of which he carried out several population based etiological studies on tobacco, alcohol, occupation and environmental pollution. As soon as the registry started producing survival data he promoted the **EUROCARE** project, a concerted action between virtually all European cancer registries aimed at describing survival differences, understand the reasons why they exist, and help preventing inequalities (*Berrino et al Survival of Cancer Patients in Europe, IARC Scientific Publications 132, 1995, and 151, 1999; Annals of Oncology (supplement 5), 2003*). Over 100 scientific publications ensued, the most recent ones on survival of patients diagnosed in the late 1990s and early 2000s (*Berrino et al. 2007 Lancet Oncology 8:773*), which influenced National Cancer Plans in several European countries. FB was rapporteur on cancer patients survival for the Slovenian EU Presidency book reviewing the status of cancer control in Europe, published at the Ljubljana cancer conference in February 2008 (*'Responding to the challenge of cancer in Europe'*). He also promoted the comparison of cancer patients survival in Europe and the USA (*Gatta et al Cancer 2000 89:893, and 2002, 95:1767; Sant et al. Cancer 2004, 100:715; Ciccolallo et al. Gut 2005, 54:268; Coleman et al 2008 Lancet Oncol 9:730*) and showed that the differences are largely due to earlier diagnosis in the US, and not to stage specific more effective treatment.

In the 1980s FB planned and implemented the first Italian prospective cohort study with biological bank of serum, plasma, WBC, RBC and overnight 12h urine collected according to highly standardized procedures: the **ORDET** study on hORmones and Diet in the Etiology of Tumours, based on 11,000 healthy women residing in the area covered by the Varese Province Cancer Registry. Major ORDET results include the significant positive association of breast cancer with pre-diagnostic serum concentration of androgens (both after menopause, *Berrino et al. 1996 JNCI 88:29*, and before menopause, *Micheli et al 2005 Int J Cancer 112:312*), glucose, IGF-I (*Muti et al. 2002 Cancer Epidemiol Biomarkers Prev 11:1361; Sieri et al. 2012 Int J Cancer 130:921*), low urinary 2:16alpha-hydroxyestrone ratio (*Muti et al. 2000 Epidemiology 11:635*), low urinary 6-sulfatoxymelatonin (*Schernhammer et al. 2008 JNCI 100:898*), with the metabolic syndrome (*Agnoli et al. 2010 Nutr Metab Cardiovasc Dis 20:41.*), and with several dietary factors: saturated fat, glycemic load , and Mediterranean dietary pattern, specially protective for HER2+ tumours (*Sieri et al. 2002 Nutr Cancer 42:10; 2004 Cancer Epidemiol Biomarkers Prev 13:567; 2007 Am J Clin Nutr 86:1160; Sant et al. 2007 Int J Cancer 121:911*). ORDET contributed to the pooled analysis of several cohort studies that eventually confirmed the association of IGF-I and sex hormones with breast (*Key et al. JNCI 2002, 94:606; Crowe et al 2009 Cancer Epidemiol Biomerkers Prev 18:1333*) endometrial and ovarian cancer (*Lukanova et al 2002-3-4 Int J Cancer 108:262; 108:425; 104:636; 101:549*).

The ORDET experience was pivotal to the development of the **EPIC** (European Prospective Investigation into Cancer and nutrition) multinational cohort, the largest prospective study with biological repository ever undertaken. FB co-authored several EPIC papers on diet and head and neck, stomach, colon, lung, breast, endometrium, ovary, prostate, and bladder cancer in relation to diet, anthropometric and metabolic and hormonal factors. Given this long lasting experience, FB co-ordinated the section on breast, ovary and cervix cancer for the American Institute for Cancer Research / World Cancer Research Fund systematic literature review (*AICR/WCRF Food, Nutrition, Physical Activity and the Prevention of Cancer: a Global Perspective, 2007*)

In the 1990s FB realized that the accumulated evidence on hormones, lifestyle and breast cancer was sufficiently strong to justify the undertaking of randomized dietary intervention studies on women at high risk because of high serum androgen levels or other endocrine-metabolic high risk traits. The rationale was that western sedentary and hyper-caloric diet lifestyle is associated with insulin resistance, and insulin stimulates the synthesis of androgens in the ovary and inhibits the liver synthesis of SHBG and IGFBP1 and 2, thus increasing the bioavailability of both sex hormones and IGF-I. The **DIANA** (DIet and ANdrogens) trials proved that a comprehensive dietary modification aiming at lowering insulin levels, based on Mediterranean traditions and macrobiotic principles (low fat, refined carbohydrates and animal products, high whole grain cereals, legumes and vegetables) decreases the bioavailability of sex hormones and several growth factors (*Berrino et al. 2001 Cancer Epidemiol Biomarkers Prev 10:25, and 2006 Ann NY Acad Sci 1089:110*), and suggested that can decrease breast cancer recurrences (*Berrino et al. 2005 Int J Cancer 113:499*). In the latter study high serum testosterone levels, high BMI, the presence of metabolic syndrome *and the contemporary presence of high* IGF-I and PDGF *were significant negative prognostic factors* (*Pasanisi et al. 2006 Int J Cancer 119:236, and 2008 Cancer Epidemiol Biomarkers Prev, 17:1719*). Actually the same hormonal and metabolic indicators of breast cancer risk also appeared to effect breast cancer prognosis. The success of the DIANA studies, which were based on a few hundred patients, prompted the planning of a large scale randomized trial of adjuvant diet and physical activity to reduce recurrences in

breast cancer patients with endocrine/metabolic pattern suggesting high recurrence risk (either high testosterone, or insulin, or presence of metabolic syndrome). The trial has recruited 2000 patients and the follow-up is ongoing (*Villarini, et al, Lifestyle and breast cancer recurrences: The DIANA-5 trial. Tumori, 98: 14-31, 2012).*

FB has no interests of any kind that could reasonably be perceived to affect his scientific objectivity. His scientific activity is motivated solely by his passion for cancer prevention.

Funding ID

There is and there will be no funding overlap with the ERC grant requested and any other source of funding for the same activities and costs that are foreseen in this project.

In relation to this proposal FB intends to apply for a further grant to study epigenetic profiles before and after calorie restriction and metformin treatment. Such a study would involve only a few hundred persons volunteering for the present MeMeMe project, and they will be requested to sign an independent informed consent. A previous application to the Italian CARIPLO Foundation failed; the following box reports its executive summary:

Potentially preventive epigenetic changes functionally related with calorie restriction and metformin treatment

The western life style, characterized by a hyper-caloric diet rich in fat, refined carbohydrates and animal proteins is associated with a high prevalence of obesity, diabetes,metabolic syndrome (MetS), insulin resistance, and elevated haematic levels of growth factors and, in women, sexual hormones. These factors are also associated with a high risk for some of the most frequent tumours in western population, such as colorectal and breast cancers (CRC and BC), and to a higher relapse risk in patients that have already been diagnosed and treated for these diseases. Dozens of animal experiments have proven beyond reasonable doubt that moderate calorie restriction without malnutrition (CR) is the most potent dietary intervention for improving insulin sensitivity and other markers of MetS, preventing cancer, and prolonging life. A sustainable CR can be obtained with traditional Mediterranean diet (MedDiet), characterized by highly satiating staple food, such as whole grain cereals and pasta, legumes, seasonal vegetables, nuts, olive oil, fruit, moderate wine, and occasionally animal products, mainly fish. MedDiet prevents MetS and is associated with lower incidence of several cancers, including BC and CRC. Diabetic patients have increased risk of cancer, but several observational studies showed a significantly lower risk among those treated with metformin (MET), a CR mimetic.

There is increasing preclinical evidence suggesting that CR and MET favourably modify the risk of cancer through epigenetic mechanisms. We intend to explore such mechanisms in humans. We will profit of our large prospective cohort studies with biological repository (plasma, serum, buffy coat), in which we are following up tens of thousands of healthy women for the occurrence of cancer, of our clinical cohorts of thousands of BC patients, and of our randomized controlled studies of dietary intervention and chemoprevention, in which we are treating volunteers, either healthy but with MetS (for the prevention of cancer), or with previous diagnosis of BC (for the prevention of metastasis), with either CR or MET.

We will analyse epigenetic traits (DNA methylation, histone modification, and miRNA expression) before and after treatment with CR, MET, or both, in women with or without MetS, abdominal obesity, and different lifestyle and dietary habits. We will then evaluate if the epigenetic changes discovered in women enrolled in our dietary and chemopreventive trials are associated with the subsequent incidence of BC and CRC in our epidemiological cohorts, and with the occurrence of metastases in our clinical cohorts (taking into account in the study design and in the analysis the classical epidemiological and clinico-pathological risk factors). We will examine in particular the incidence of metastasis in women with triple-negative BC. In a sample of women we will study the epigenetic traits in epithelial breast cells obtained through nipple aspiration.

As it recently emerged that MET can directly suppress the proliferation of tumour initiating cells (TICs or cancer stem cell), we will also test this hypothesis in vivo treating with MET immunodeficient mice xenotransplanted with human CRC cells enriched in TICs, and we will analyse the induced epigenetic changes, which will be than compared with that observed in women subjected to the different treatments.

The new study we intend to apply for is not a human gene-mapping study; we are not interested in identifying any allele that predispose to an illness, nor in carrying out family studies; we have no commercial interest nor partners. It is just a study on the epigenetic effects of calorie restriction and of treatment with metformin to be carried out on a voluntary sample of participants in the main study. Depending on the discoveries of such epigenetic research, however, the analysis of a few specific genes, e.g. genes whose variants may affect the effectiveness of calorie restriction or metformin, such as AMPK and LKB1, may become relevant to the interpretation of the results. We will describe in terms understandable to the participants the nature of this new epigenetic/genetic study, in order to give them an opportunity to decide whether or not to participate. We will guarantee that all studies involving their samples will be done anonymously, and that no one outside the

study team will have access to the individual data. We will respect participants' decision to consent or not consent the use of their samples beyond the primary objective of exploring the epigenetic changes. In case they accept we will be available to inform them on the significance of the genetic traits that may emerge.

10-Year-Track-Record

Major studies published in the years '2000 with Dr. Berrino as senior author (either first or last name).

Preventing weight gain during adjuvant chemotherapy for breast cancer: a dietary intervention study.
Villarini A, Pasanisi P, Raimondi M, Gargano G, Bruno E, Morelli D, Evangelista A, Curtosi P, Berrino F.
Breast Cancer Res Treat. 2012 Sep;135(2):581-9. doi: 10.1007/s10549-012-2184-4

Effect of different doses of metformin on serum testosterone and insulin in non-diabetic women with breast cancer: a randomized study.
Campagnoli C, Pasanisi P, Abbà C, Ambroggio S, Biglia N, Brucato T, Colombero R, Danese S, Donadio M, Venturelli E, Zito G, Berrino F.
Clin Breast Cancer. 2012 Jun;12(3):175-82

Burden of testicular, paratesticular and extragonadal germ cell tumours in Europe.
Trama A, Mallone S, Nicolai N, Necchi A, Schaapveld M, Gietema J, Znaor A, Ardanaz E, Berrino F; RARECARE Working Group.
Eur J Cancer. 2012 Jan;48(2):159-69

Lifestyle and breast cancer recurrences: The DIANA-5 trial
Villarini A, Pasanisi P, Traina A, Mano MP, Bonanni B, Panico S, Scipioni C, Galasso R, Paduos A, Simeoni M, Bellotti E, Barbero M, Macellari G, Venturelli E, Raimondi M, Bruno E, Gargano G, Fornaciari G, Morelli D, Seregni E, Krogh V, and Berrino F
Tumori, 98: 14-31, 2012

Serum levels of IGF-I and BRCA penetrance: a case control study in breast cancer families.
Pasanisi P, Bruno E, Venturelli E, Manoukian S, Barile M, Peissel B, De Giacomi C, Bonanni B, Berrino J, Berrino F.
Fam Cancer. 2011 Sep;10(3):521-8.

Menopausal hormone therapy and breast cancer risk: Impact of different treatments. The European Prospective Investigation into Cancer and nutrition (EPIC).
Bakken K, Fournier A, Lund E, Waaseth M, Dumeaux V, Clavel-Chapelon F, Fabre A, Hémon B, Rinaldi S, Chajes V, Slimani N, Allen NE, Reeves GK, Bingham G†, Khaw K-T, Olsen A, Tjønneland A, Rodriguez L, Sánchez M-J, Etxezarreta PA, Ardanaz E, Tormo M-J, Peeters PH, van Gils CH, Steffen A, Schulz M, Chang-Claude J, Kaaks R, Tumino R, Gallo V, Norat T, Riboli E, Panico S, Masala G, González CA, Berrino F.
Int J Cancer. 2011 Jan 1;128(1):144-56.

Trends of nutritional epidemiology in Europe.
Berrino F.
Epidemiol Prev. 2010 Sep-Dec;34(5-6):15-8.

Oral contraceptive use and BRCA penetrance: a case-only study.
Pasanisi P, Hédelin G, Berrino J, Chang-Claude J, Hermann S, Steel M, Haites N, Hart J, Peled R, Gafà L, Leggio L, Traina A, Amodio R, Primic-Zakelj M, Zadnik V, Veidebaum T, Tekkel M, Berrino F.
Cancer Epidemiol Biomarkers Prev. 2009 Jul;18(7):2107-13.

Serum insulin-like growth factor-I and platelet-derived growth factor as biomarkers of breast cancer prognosis.
Pasanisi P, Venturelli E, Morelli D, Fontana L, Secreto G, Berrino F.
Cancer Epidemiol Biomarkers Prev. 2008 Jul;17(7):1719-22

Survival for eight major cancers and all cancers combined for European adults diagnosed in 1995-99: results of the EUROCARE-4 study.
Berrino F, De Angelis R, Sant M, Rosso S, Bielska-Lasota M, Coebergh JW, Santaquilani M; EUROCARE Working group.
Lancet Oncol. 2007 Sep;8(9):773-83

Serum testosterone levels and breast cancer recurrence.
Berrino F, Pasanisi P, Bellati C, Venturelli E, Krogh V, Mastroianni A, Berselli E, Muti P, Secreto G.
Int J Cancer. 2005 Jan 20;113(3):499-502.

Endogenous sex hormones and subsequent breast cancer in premenopausal women.
Micheli A, Muti P, Secreto G, Krogh V, Meneghini E, Venturelli E, Sieri S, Pala V, Berrino F.
Int J Cancer. 2004 Nov 1;112(2):312-8.

Fasting glucose is a risk factor for breast cancer: a prospective study.
Muti P, Quattrin T, Grant BJ, Krogh V, Micheli A, Schünemann HJ, Ram M, Freudenheim JL, Sieri S, Trevisan M, Berrino F.
Cancer Epidemiol Biomarkers Prev. 2002 Nov;11(11):1361-8.

Reducing bioavailable sex hormones through a comprehensive change in diet: the diet and androgens (DIANA) randomized trial.
Berrino F, Bellati C, Secreto G, Camerini E, Pala V, Panico S, Allegro G, Kaaks R.
Cancer Epidemiol Biomarkers Prev. 2001 Jan;10(1):25-33

Erythrocyte membrane fatty acids and subsequent breast cancer: a prospective Italian study.
Pala V, Krogh V, Muti P, Chajès V, Riboli E, Micheli A, Saadatian M, Sieri S, Berrino F.
J Natl Cancer Inst. 2001 Jul 18;93(14):1088-95.

Research monographs on cancer survival:

Survival of cancer patients in Europe. The EUROCARE Study.
Berrino F, Sant M, Verdecchia A, capocaccia R, Hakulinen T, Estève J.
IARC Scientific Publications N° 132 IARC Press, Lyon, France, 1995

Survival of cancer patients in Europe. The EUROCARE-2 Study.
Berrino F, Capocaccia R, Estève J, Gatta G, Hakulinen T, Micheli A, Sant M, Verdecchia A.
IARC Scientific Publications N° 151 IARC Press, Lyon, France, 1999

Survival of cancer patients in Europe. The EUROCARE-3 Study. Berrino F, Capocaccia R, Coleman MP, Estève J, Gatta G, Hakulinen T, Micheli A, Sant M, Verdecchia A.
Annals of Oncology, Volume 4, Supplenment 5, 2003

Comparative cancer survival information in Europe.
Berrino F, Verdecchia A, Lutz JM, Lombardo C, Capocaccia R.
In: Survival of cancer patients in Europe, 1995-2002: The EUROCARE-4 Study.
Capocaccia R, Gavin A, Hakulinen T, Lutz JM, Sant M (eds).
European Journal of Cancer, Vol 45, No 6, 2009 (Special issue)

Dr Berrino has been invited several times to international conferences (UICC Int Cancer Congresses, ICCC, ECCO, ESMO, EACR, AICR-WCRF, IACR, GRELL, CR-UK, EU parliament...) and advanced schools (IARC Epidemiology courses, ESO, LSHTM...)

a. Background and ol

Worldwide, we are entering a period of unprecedented global aging, and age-related chronic non-communicable diseases **(ArCD)** and related disabilities are projected to inflate into an unprecedented economic and social challenge. ArCD are largely due to modifiable western lifestyle factors. Previous lifestyle and chemopreventive trials to prevent ArCD have generally been unsuccessful. Most were based on the supplementation or the avoidance of a single or a few nutrients, and most failed or ended with dubious results. The ATBC, CARET and SELECT trials, which supplemented antioxidant vitamins or minerals to prevent cancer, were interrupted because of increased cancer incidence or serious side effects. Also in the cardiovascular field preventive trials supplementing antioxidant and other vitamins showed little or no cardiovascular benefit. Nevertheless, the present epidemiological and biological knowledge of ArCD makes a new generation of preventive trials promising, feasible, and worthwhile. Progress in ArCD biology has been extremely fast in the last few decades, pointing out to the relevance of complexity as opposed to the previously dominant reductionist view of nature. Emerging data demonstrate that the effect of lifestyle factors is largely mediated by epigenetic changes, with a cross-talk that is largely to be unravelled between DNA methylation, histone modifications, and noncoding RNA. ArCD share several risk factors – overeating, sedentary lifestyle, industrially processed food, excess body weight, insulin resistance, chronic inflammatory status, and a predisposing (e.g."thrifty") genotype – and we hypothesize that a many faceted intervention is needed to favourably effect the complex biological system involved in their development. A single agent or factor acting on a single or a few pathways might actually fail because of the existence of redundant vicarious pathways, or even be dangerous because of its interference with potentially preventive pathways.

The aim of the present study is to evaluate the effect of a comprehensive life-style intervention (including moderate physical activity and Mediterranean/macrobiotic diet with moderate calorie and protein restriction), and of treatment with Metformin (a calorie restriction mimetic drug) for the prevention of ArCD. The plan is to carry out a randomized controlled trial on men and women, 55-74 years of age, at high risk of ArCD because of metabolic syndrome.

Metabolic syndrome (MetS)

The major metabolic risk factor for the development of ArCD is the MetS, whose prevalence in adult population ranges between 20 and 40% in different western countries. MetS is defined as a clustering of cardiovascular disease (CVD) risk factors of metabolic origin. The World Health Organization (WHO) was the first to propose criteria for diagnosis of the syndrome, followed by several other organizations. The most frequently used criteria are those of the National Cholesterol Education Program Adult Treatment Panel III (*2001 Jama.285:2486*), which defines MetS as the presence of three or more of the following conditions: Waist circumference equal to or greater than (≥) 88 centimeters in women or 102 cm in men , Glucose ≥ 5.6 (mmol/l), Triglycerides ≥ 1.7 mmol/l, HDL-Cholesterol <1.0 (men) or <1.3 mmol/l (women), blood pressure ≥ 130/85 mmHg. Several other thresholds were subsequently proposed by other agencies. The international Diabetes Federation, for instance, recommended that the threshold of waist circumference to define abdominal obesity in people of European origin should be ≥ 80cm for women and ≥ 94 cm for men. To simplify the instruction for the self-selection of participants our threshold for waist circumference will be 85 cm for women and 100 cm for men. As suggested in a meeting of several major organizations in an attempt to unify criteria we will consider as MetS components also the presence of specific treatments for lipid metabolism and for previously diagnosed hypertension, and the cut point for elevated fasting glucose will be ≥100 mg/100mL (*Harmonizing the Metabolic Syndrome 2009 Circulation 120:1640*).

Classical studies showed that the MetS is accompanied by a two-fold increase in the risk of CVD and a five-fold increase in the risk of type 2 diabetes mellitus. More recently several studies showed that MetS is also associated with increased risk of cancer, neurodegenerative diseases, non-alcoholic fatty liver disease and cirrhosis, chronic kidney disease, gallbladder stones, kidney stones, osteoarthritis, gout, prostatic hyperplasia, thromboembolic disease, psoriasis and other chronic conditions. MetS related cancers include colorectal, liver, gallbladder, pancreas, breast, cervix, endometrium, ovary, kidney, esophagus (adenocarcinomas), and probably prostate and lung cancer, representing two thirds of total cancer incidence. The mechanism is likely to involve hyperglycemia, hyperinsulinemia, and its effect on the synthesis and bioavailability of sex hormones, growth factors, and proinflammatory cytokines. Increased leptin and decreased adiponectin levels also disrupt homeostatic signaling pathways involved in cell proliferation, survival, cell-cycle regulation, and angiogenesis.

Calorie restriction (CR)

Since the beginning of the last century, moderate CR without malnutrition (an experimental mode in which test animals receive a lower-calorie diet than *ad libitum*-fed controls) has emerged as the most potent dietary intervention for prolonging life and preventing ArCD, including cancer, diabetes, artheriosclerosis, cardiomyopathies, autoimmune diseases neurodegenerative diseases, respiratory and kidney diseases. Results in rhesus monkeys also showed that Sarcopenia, a serious health concern associated with advancing aging, was partially prevented in the CR group, as well as the loss of Tcell function and gray

matter volume. Humans undergo several of the same adaptations i animals, including lower levels of cardiovascular risk factors, inflammatory cytokines, thyroid hormones, insulin, leptin, sex hormones (in women), and higher levels of steroid hormone-binding proteins, adiponectin and other hormones that suppress inflammation, better left ventricular diastolic function, and lower carotid intima-media thickness than healthy age and sex matched controls. Recently, CR and a pharmacological CR mimetic (rapamycin, a macrolide that produces a gene expression pattern that overlap that seen with CR) were shown to extend lifespan and delay cancer incidence in mice that were already in late middle age when treatment began (up to 19 months, suggesting that starting lifestyle interventions on men and women having broadly the same age – above 55 years or so – could be promising).

The responses to CR involve numerous pathways and intricately linked mechanisms that are far from being completely understood. Among the mechanisms by which CR may reduce metabolic and neoplastic risk the well established ones are:

A) the decrease of the ATP/ADP ratio, with the subsequent activation of the adenosine 5'-monophosphate-activated protein kinase (AMPK), a sensor of energy availability (the "fuel gauge"), whose activation inhibits energy consuming processes, including cell proliferation, and activates various other potentially preventive pathways.

B) the decrease of the $NADH/NAD^{+}$ratio, followed by NAD dependent deacetylases (sirtuins) activation, which epigenetically regulate gene expression (e.g. of FDXO, p53, NF-kB).

and C) the downregulation of the endocrine Insulin/IGF-1 pathway, and the nutrient-responsive kinase target of rapamycin (TOR).

Various posttranscriptional alterations relevant to CR are being discovered, e.g. miR-217, which inhibits SIRT1, and promoter methylation profile of leptin and TNF-alpha during weight loss.

Several studies suggested that most of the effect of CR is due to protein restriction, which seems necessary, at least in humans, to reduce the plasma concentration of IGF-I, whose high level is associated with increased risk of subsequent cancer. In western countries people eat about the double of the RDA of proteins (15-16% of calories vs. 8.3%), and reducing protein intake may be more sustainable than reducing total calorie intake. Both objectives, however, can be obtained with traditional Mediterranean diet and with macrobiotic diet.

Mediterranean diet (MedDiet)

Classical studies showed that MedDiet (i.e. a diet that was largely based on unrefined cereal products (mainly bread and pasta), pulses, vegetables, olive oil, nuts, fruit, moderate wine, occasionally fish and cheese, and rarely other animal products) is associated with lower risk of CVD and diabetes. Subsequently it became clear that subjects with the highest adherence to a traditional MedDiet have lower cumulative incidence of MetS, and of several ArCD than those with the lowest adherence. Randomized intervention trials showed that MetS can be reversed with MedDiet, with up to 69% reduction of MetS prevalence after 2 years of diet. Several other trials supported favourable effects of MedDiet on body weight, abdominal adiposity, plasma lipid profile, and endothelial function. Lower adherence to MedDiet is also associated with the development of cognitive impairment and of Parkinson and Alzheimer disease.

Several observational studies on dietary patterns showed that MedDiet is significantly associated with lower risk of breast cancer, colon cancer, melanoma, coronary hearth disease), stroke, diabetes, Alzheimer disease, and overall mortality.

Whole grain cereal intake and macrobiotic diet

Several studies showed consistently that persons who ingest three or more portions of food per day based on wholegrain cereals and whole-wheat bread have a 20-30% lower risk of cardiovascular diseases and type-2 diabetes than subjects who ingest low quantities of cereals. Two recent large cohort studies on the intake dietary fibres and mortality, the EPIC study in Europe and the NCI-ARP study in the US, consistently showed that an high intake of cereal fibres is associated with lower mortality from cancer, cardiovascular, pulmonary, gastrointestinal, and infectious diseases. The isolated administration of whole grain cereal components (fibres, vitamins, minerals), or the same amount of fibre from refined grains, do not seem to elicit any protective effect, suggesting additive or more than additive influence of cereal constituents on health, the so called "food synergy" Whole grain cereals (mainly brown rice, millet, barley, oats) and occasionally buckwheat and wheat pasta, are the basic component of macrobiotic diet, which includes also 20–30% locally grown vegetables, 5–10% beans, including traditional soy products, sea vegetables and occasionally fruits, nuts, fish. The 2007 WCRF/AICR recommendations for the prevention of cancer (www.dietandcancerreport.org) broadly coincide with the macrobiotic recommendations of avoiding the habitual intake of extremely "yin" food, such as sugared and alcoholic beverages, as well as of extremely "yang" food, such as processed meat, salty food and red meat, while the central recommendation is to "Eat mostly food of plant origin, with a variety of non starchy vegetables and of fruit every day and with unprocessed cereals and/or pulses within every meal", which is also the basic characteristic of the MedDiet, *as well as the staple food of most populations before the industrial revolution: cuscus with chickpeas in north*

Africa, rice with soy products in Asia, maize with black beans ir ... ›anuts in black Africa.

Our dietary randomized controlled trials in postmenopausal women (the DIANA - Diet and Androgens - trial) showed that a comprehensive dietary modification, based on macrobiotic principles and MedDiet, elicited a very high compliance and dramatically improved, over a few months, the hormonal and metabolic pattern associated with MetS and breast cancer risk, decreasing body weight (4 Kg), waist circumference (4 cm), glycaemia (-6%), triglycerides (-15%), total cholesterol (-14%) , testosterone (-18%), free estradiol (-23%), and insulin (-10%), and increasing sex hormone-binding globulin (SHBG, +25%) and insulin-like growth factor-binding protein (IGFBP-1, +12%; and IGFBP-2, +25%), thus decreasing the bioavailability of sex hormones and IGF-I. Such favourable effects may have been largely due to the moderate CR that we obtained reducing highly processed calorie-dense food and refined carbohydrates and increasing the consumption of highly satiating unrefined cereals, legumes and vegetables. Adjusting for weight loss, in fact, the metabolic effects tended to lose statistical significance.

Physical activity

In observational studies, moderate physical activity is associated with lower incidence of CVD, cancer, lower prevalence of MetS parameters , and improved diabetes control. Several randomised studies of lifestyle education that included both dietary modification (mainly calorie restricted, low fat, and high fibre diets) and physical exercise consistently showed a 50% lower incidence of diabetes in persons with impaired glucose tolerance or impaired fasting glucose.

Metformin (MET)

MET, an orally administered biguanide drug widely used to treat diabetes, has been shown to prevent diabetes in people with glucose intolerance, to reduce cardiac risk factors and prevent CVD in diabetics, and to improve clinical outcomes in patients with heart failure. Improved glycemic control in patients treated with metformin is associated with a decreased risk of diabetes related cardiovascular end points and all cause mortality when compared to conventional therapies that lower blood glucose to similar levels. Therefore, the cardioprotective effects of metformin cannot be attributed to its anti-hyperglycemic effects alone, but may be also related to the actions of metformin on lipid metabolism, vascular smooth muscle proliferation, cardiomyocyte calcium handling, endothelial function, hypercoagulation , and platlet reactivity. Such pleiotropic effects of metformin are largely mediated by the activation of AMPK, a protein that is normally activated in response to CR, which stimulates fatty acid oxidation, promotes glucose transport and glycolysis, inhibits neoglucogenesis and both triglycerides and protein synthesis.

Diabetes is associated with increased cancer incidence. However, several observational studies (mainly retrospective cohort studies) showed that diabetic patients treated with metformin have a significantly lower risk of developing cancer (all sites combined) than those untreated or treated with other drugs. MET may reduce cancer risk through two main mechanisms:

1st) reducing insulin resistance and therefore insulin, testosterone and IGF-I concentration in blood, and

2nd) activating AMPK, thus mimicking the effect of CR. AMPK activation results in downregulation of mTORC1 and IGF1/Akt pathways, and in p53-mediated cell cycle arrest. MET also inhibits IRS-1, thus preventing the feed-back activation of Akt that may follow mTOR inhibition.

In a phase-2 randomized study we recently showed that 1500 mg/day of metformin for 5 months significantly decreases body weight (-2.8 kg, $P<0.01$), and blood levels of glucose and insulin, improving insulin sensitivity (HOMA-IR index -0.23, $P = 0.02$), while 1000 mg/day elicit only minor effects. In this study MET1500mg also reduced the plasma concentration of testosterone (-0.07 ng/mL, $P<0.01$), and increased SHBG (+10,6 nmol/L, $P<0.01$), which in older women are associated with insulin resistance, metabolic syndrome, CVD and breast cancer. Estradiol also decreased significantly in women who lost weight.

MET is a very safe drug used since 50 years to treat diabetes and more recently to prevent diabetes in people with glucose intolerance. As phenformin, a drug acting through the same mechanism, caused several cases of lactic acidosis, the issue was raised that also MET might cause such a serious side effect. As stated in a recent Cochrane systematic review, however, "There is no evidence from prospective comparative trials or from observational cohort studies that metformin is associated with an increased risk of lactic acidosis, or with increased levels of lactate" (*Cochrane Database Syst Rev. 2006 Jan 25;(1):CD002967*). Nevertheless we will carefully exclude from the study any person with chronic hypoxemic conditions that may be associated with lactic acidosis, such as cardiovascular, renal, hepatic and pulmonary disease, or under treatment with drugs that might interfere with MET clearance (see operative protocol). A physician will be available 24h/24 to answer questions on the phone if the participants do not feel well for whatever reason.

Molecular pathways that mediate the preventive effects of CR and of MET

Specific DNA methylations and histone modifications, usually associated with pro-inflammatory micro-environment have been implicated in several ArCD (cancer, CVD, obesity, COPD). Global hypomethylation and site-specific hypermethylation are common features of human tumours and have been detected also in atherosclerotic lesions. Diet can profoundly alter epigenetic patterns, but the causal link between diet and epigenetics in the development of human disease is still poorly understood. A challenging research field is developing to determine which adverse epigenomic marks are reversible by specific drugs, nutrients, or lifestyle changes. Besides CR, over 50 bioactive phytochemicals have been demonstrated to be active on DNA methyltransferase, or histone acetylase/deacetilase. Experimental studies on animals or cultured human cell lines support their role in the prevention of ArCD, but have often been conducted at concentrations far beyond those documented in humans. It is very difficult to predict from these results the effects of these substances on disease prevention in humans. There is increasing evidence, however, that continuous exposure at physiological concentrations can remodel the epigenome in a cumulative fashions. We will create a biorepository of blood samples, collected at two time points (before randomization, and after one year), to carry out mechanistic studies on the epigenetic changes induced by lifestyle modification and MET treatment. With independent financial support we intend to systematically explore the epigenetic changes induced by CR, MET and CR + MET. A financial request has been submitted to the Italian CARIPLO Foundation (see Funding ID).

Importance of the project

Our important, innovative, and bold hypothesis is that we can prevent, or at least postpone, the occurrence of virtually all ArCD through a comprehensive change of several aspects of western lifestyle, supported by a chemopreventive agent that acts on the same genetic pathways affected by lifestyle factors, and that we will obtain a very high compliance. Our confidence derives from the metabolic success of previous dietary intervention studies on a few hundred subjects (the DIANA trials) and from the preliminary results of our ongoing DIANA-5 trial, in which we succeeded to recruit 2000 breast cancer survivors that have been randomized in a lifestyle intervention and a control group to study the effect of moderate CR and increased physical activity on the incidence of recurrences

Changing so many aspects of lifestyle, and examining so many outcomes, we will not be able to understand what has caused what. This may be irrelevant from a pragmatic public health point of view, but is certainly relevant from an explicative point of view. Beside the basic "intention to treat" analysis of the trial, therefore, we will analyse the outcomes also as a function of compliance with the different lifestyle recommendations, and of their association with MET treatment. With repeated 24h food diaries, for instance, we will be able to disentangle the role of protein restriction from that of CR, and to explore the effect of specific foods. Results will not have the strength of a randomized design, but will help mechanistic interpretation. Continuing the observation beyond the presently planned 5 years we will also have the power to study several specific diseases.

b.

Methodology

Main study hypothesis

Our hypothesis is that a substantial fraction of ArCD incidence and mortality, of the order of 25-33%, could be prevented through a) a sustainable comprehensive change in lifestyle, including moderate calorie and protein restriction, Mediterranean/macrobiotic diet, and physical activity, and b) chemopreventive treatment with metformin, a drug whose mechanism include the activation of the same genetic pathways that are activated by CR and physical activity. An ancillary study will examine the epigenetic changes induced by the above treatments.

Study design

Phase III randomized controlled trial on men and women with MetS to test the hypothesis that comprehensive life-style changes and/or metformin treatment prevent ArCD.

Design: 2x2 factorial: 2,000 volunteers will be randomized in four equal groups of 500 each, and allocated to the following treatments:

- METFORMIN (1700MG/DAY) + ACTIVE LIFESTYLE INTERVENTION
- PLACEBO + ACTIVE LIFESTYLE INTERVENTION
- METFORMIN (1700 mg/day) alone
- PLACEBO alone

The metformin /placebo component of the study will be double blind.

Main outcome: Total incidence of ArCD, including cancer, coronary heart disease, stroke, diabetes. Secondary outcomes: changing prevalence of MS and its metabolic and anthropometric components, total mortality, total cancer incidence, incidence of diabetes, cardiovascular diseases, myocardial infarction, stroke. These outcomes have been selected as major objectives because they are fairly easily identifiable on clinical charts according to standardized criteria. Other MetS associated ArCD, however, will be registered in

the follow-up and may eventually contribute to the overall disease burden to be evaluated.

Recruitment: Potentially eligible persons will be recruited through cancer screening programmes, general practitioners, clinical units dealing with overweight and metabolic diseases, and the media. Interested persons will be invited to attend conferences in which we will illustrate in detail the study design and its implications. Paper tape measures will be available at the conference to help attendees to check their waist circumference, which is the basic eligibility criteria. Those above the threshold and without any obvious reasons not to be included, will be asked to sign the informed consent form, and will be given an appointment for the first clinic visit and blood sample donation.

Inclusion criteria:

Age 55-74, waist circumference equal or greater than 85 cm for women and 100 cm for men, plus at least two other factors among those defining the MetS

Exclusion criteria:

- Diagnosed diabetes (or baseline fasting glycemia above 7mmol/L at baseline examination)
- Cancer (except skin carcinoma) diagnosed in the last 5 years, or under treatment
- Excessive frailty: in absence of agreed-upon measurements parameters and cutoff points, we will exclude subjects under the lower 5th percentile of the muscular mass distribution estimated by impedance in previous studies
- Conditions that contraindicate the use of MET because might favour lactic acidosis
  - Renal, cardiac, hepatic, or respiratory insufficiency
  - Serum creatinine <124μmol/L, or proteinuria at baseline examination
  - Current treatment with K-sparing diuretics, or with proton pump inhibitors
  - Excessive alcohol consumption
- Distressing side effects of MET treatment. Nausea and diarrhoea typically occur in about one third of patients receiving MET for the first time at full dose. To avoid dropouts for gastrointestinal discomfort we will treat all volunteers with half the planned dose for one month in order to exclude intolerant subjects before randomization. Participants randomized in the intervention group will continue to take half a dose for one month and then shift to the full dose.

Baseline requirements

Participants will be requested:

- to sign the informed consent to participate in the study and to follow the prescribed treatment and lifestyle changes, to provide information on their health status and intervening diseases, and to allow the study officials to contact their treating physicians, to consult clinical notes and to examine blood samples for metabolic, genetic and epigenetic studies, even after the end of the active intervention period
- to fill in a questionnaire on medical history and risk factors (the EPIC-Italy questionnaire)
- to undergo an anthropometric visit (height, weight, waist and hip circumference, blood pressure, body fat and lean mass measured with Tanita impedance device, at baseline and after 1 year)
- to donate a 20 ml blood sample (at baseline and after 1 year)
- to fill in 24h dietary and exercise recall forms (either directly or on the phone) every 2 months in randomly chosen days.

Participants will be informed that they can retire from the study at any moment, but recommended not to do so because it would damage the interpretation of results.

Randomization

As soon as all the baseline data are entered, and the eligible participant confirms his/her willingness to go on, a computer system randomly establishes the study groups, balanced for gender and age (≤ 65 years vs. >65 years), and gives instruction to the pharmacy to prepare the drug bottles. The assignment is immediately recorded in the database and not subject to alteration.

Prescriptions:

A) 2 tablets per day, one at breakfast (or lunch) and one at dinner, of either metformin (two 850 mg tablets/day) or placebo (two identical tablets) according to the blind assignment. In case of gastrointestinal discomfort occurring after randomization participants will be asked to continue with a single tablet. In our pilot trial such side effect occurred in 10 out of 80 participants: 7 of them stopped taking the pill and 3 continued at half the dose. Subjects stopping taking the pills will be kept in the study, but we will increase the study size by 10% to partly compensate the power loss.

B) participation in the life-style intervention activities. All participants will receive general lifestyle recommendations for the prevention of cancer and cardiovascular diseases, including smoking cessation counselling, and those randomized in the life-style intervention group will be offered to participate to four kitchen courses and will then be invited monthly for lunch or dinner, physical activity sessions, and nutritional counselling to strengthen their commitment over the whole duration of the study. Such a schedule proved to be sufficient to obtain significant reduction of body weight (3 Kg on average after one year) and of MetS parameters in our ongoing randomized trial of lifestyle modification in breast cancer patients.

Organization

We plan to enrol 25-30 volunteers per week over two years (25 persons per 40 weeks per 2 years = 2,000). Fasting blood samples will be collected between 8 an 9 a.m. Glucose, total and HDL cholesterol, and triglycerides will be immediately measured. We will then prepare 10 aliquots of serum (4 aliquots), plasma (3), red blood cells (1), buffy coat (2), to be preserved at -80°C. The blood collection protocol is compatible with future categorization of genetic and epigenetic variants (we have tested it for miRNA and DNA methylation). For a 10% random sample of participants we will extract immediately the RNA for gene expression studies.

Intervention and control groups will be separately invited every 6 months through SMS messages to refill the tablet bottle and for a short conference. In such occasion we will count the leftover tablets and participants will fill-in a questionnaire on any health events that may have occurred in the interval.

We have developed a very efficient system for communicating to study participants through SMS.

We will use the Milan national Cancer Institute facilities for blood collection and sample storage, laboratories for biochemical, and molecular biology determinations, open air physical exercise, gymnasium, teaching rooms, and dedicated kitchen and restaurant with 60 places for cooking classes and common meals.

Lifestyle intervention

The intervention aims at increasing physical activity, controlling weight, and promoting nutrient-rich (i.e. 100% RDA for all nutrients) moderately calorie-restricted and protein-restricted low-glycemic index diet.
As for physical activity, the goal of the intervention is to achieve and maintain regular participation in a moderate intensity physical activity (approximately 3 to 5 METs) program of 210 minutes over at least 3 days /week (30 min on average per day). During the first four months, one group physical activity teaching session per month will be offered. For self-monitoring and compliance enhancement, study participants will be given a stepcounter and asked to periodically fill-in 24h physical activity diaries. Persons who wish to take up vigorous sports will be encouraged to do so after cardiorespiratory fitness testing. For those who do not progress to more vigorous activity, the focus will be on maintaining moderate intensity activities, such as walking.
To control weight, participants will be encouraged to include whole grains and high-fibre vegetables, which add bulk and volume, in every meal, to choose cooked cereals rather than white bread or dry crackers, to eat vegetables or a soup before eating higher energy foods, to choose fresh fruit rather than juice, to eat nuts at least every other day, and to avoid potatoes, sugary drinks, commercial sweets, white bread and other products based on refined flours, high protein meals, and energy dense industrial foods.
In order to reduce glycaemic and insulinemic response, recommendations will include:
- reducing calorie intake, through the preferred consumptions of highly satiating foods, such as unrefined cereals, legumes and vegetables
- reducing high glycaemic index food, such as refined flours, potatoes, white rice, corn flakes, and high insulinemic foods, such as sugar and milk, preferring instead whole grain rice, barley, millet, oat, spelt, quinoa and buckwheat, legumes (any type including soy products), seasonal vegetables (any type, except potatoes)
- reducing sources of animal saturated fat (red and processed meat, milk and dairy products) preferring instead unrefined vegetable fats, such as olive oil, nuts and oleaginous seeds
- reducing protein intake, mainly animal protein (except fish), down to 10% of total calorie intake
- eat mostly food of plant origin, with a wide variety of seasonal products.

We will teach how to prepare traditional Mediterranean and macrobiotic gastronomic dishes (healthy, satiating, palatable and easy to prepare) based on whole grain cereals, durum wheat pasta, and legumes, seasoned with vegetable sauces and little fat; and competitive cakes and cookies without sugar, milk, butter, and refined flour, using instead dried fruit, such as raisin and apricots, oleaginous seeds, soy milk, cereal flakes and unrefined flour. We will use dietary sources of anti-inflammatory food, such as unrefined cereals, small size oily fish, seaweeds, turmeric, green tea, olive oil, wild berries and other quercetin rich vegetables, such as capers, apples, onion, horseradish, broccoli and other green vegetables, as well as other vegetables rich in phytochemicals that have been shown in vitro to inhibit Akt (which is critical to both cancer and atherothrombotic diseases) and other relevant genetic pathways, such as, genistein, morin, apigenin, delfinidin, luteolin. The principle, however, is to avoid any excess and to guarantee the intake of every potentially preventive bioactive phytochemicals including in the menus a wide variety of seasonal vegetables and fruits. We have recently published a book on the dietary prevention of cancer (Villarini and Allegro, Prevenire I tumori mangiando con gusto, Sperling & Kupfer 2009) which include the recipes that will be taught to the lifestyle intervention group.

Follow-up

We will actively contact the participants for the whole duration of the study, and obtain copy of relevant clinical records. The availability of an efficient regional hospital discharge diagnosis information systems, and of an accessible national death index will make follow-up feasible and complete. The Lombardy region, moreover, is almost completely covered by a network of cancer registries, which will allow to retrieve pathology reports. We will retrieve records for all ArCD but we will first concentrate the analysis on cancer, coronary heart disease, stroke, and diabetes, for which we have already used the above follow-up procedures in several previous studies. For the registration of cancer cases we will follow the rules and definitions of the European Network of Cancer Registries (available at www.iarc.fr). We will register cancer site, morphology, TNM stage, grade, and, for breast cancer hormonal receptor expression. For the definition of diabetes we will follow the criteria of the American Diabetes Association: a) Symptoms of diabetes plus casual plasma glucose concentration ≥200 mg/dl (11.1 mmol/l). Casual is defined as any time of day without regard to time since last meal. The classic symptoms of diabetes include polyuria, polydipsia, and unexplained weight loss, or b) FPG ≥126 mg/dl (7.0 mmol/l), or c) 2-h postload (75 g) glucose ≥200 mg/dl (11.1 mmol/l). d) In the absence of unequivocal hyperglycemia, these criteria should be confirmed by repeat testing on a different day.
Suspected deaths from Coronary Heart Disease (CHD) and Cerebrovascular Disease (CBVD) will be identified from mortality files when the codes ICD10 I20-I25, R96 and R98 (or E10-E14, I50, I70 in association with I20-I25), or, respectively, the codes I60-I69 (or codes E10-E14, I10-I15, I46, I49, and I70 in association with I60-I69), are reported as underlying cause of death. Fatal CHD and CBVD will be assigned only after verification against hospital discharge and clinical records.
Persons with suspected CHD or CBVD will be identified on hospital discharge forms with ICD9-CM 410-414 codes or procedure codes for coronary revascularization, and, respectively with codes 342, 430-434, 436-438 or procedure codes for carotid revascularization. Clinical records will be retrieved to verify the diagnoses. CHD will be considered verified when acute myocardial infarction, acute coronary syndrome, or coronary revascularization are present, backed up by information on onset symptoms, levels of cardiac enzymes and troponins, and electrocardiogram data coded according to the Minnesota Code. Ischemic thrombotic stroke will be diagnosed when brain infarction is mentioned in the diagnosis and/or confirmed on the basis of imaging exams (computed tomography or magnetic resonance imaging). Hemorrhagic stroke will be diagnosed when cerebral hemorrhage is mentioned in the diagnosis or confirmed by imaging.
For other potentially relevant chronic diseases, such as neurodegenerative diseases, arthritis, gout, fatty liver disease and cirrhosis, gallbladder and kidney stones, autoimmune diseases, which are not formal objectives of the application, we will examine the feasibility to collect reliable and standardized clinical diagnosis in the course of the study.

Blood examinations

At baseline and after one year we will examine the metabolic markers of MetS.
Further analysis of insulin, IGF-I, IGF-BPs, PDGF, sex hormone, hsCRP, and inflammatory cytokines, will be carried out in appropriate samples of participants from the third year onward. We will examine baseline and 1-year samples in the same batch. We will study the effect of one year CR, one year MET treatment, or their combination, in subjects with high and low compliance (defined by changes in body weight, waist circumference, and 24h recall diaries). Eventually, we will examine the change in these parameters in people who did or did not develop specific ArCD with a case control design. Changes in epigenetic profiles will be examined in the frame of companion project for which we are seeking independent financial support.

The study team has extensive experience in large-scale prospective cohort studies with biological repository (we are following-up 50,000 persons in Italy), and in dietary intervention studies (we succeeded to randomize 2,000 breast cancer survivors into a lifestyle intervention and control group and we managed to keep a very high dietary compliance of the intervention group.

Analysis

The main analyses will be by intention to treat. We will compare the incidence of ArCD in:
1,000 persons treated with MET with the 1,000 treated with placebo
1,000 persons randomized in the lifestyle intervention group with the 1,000 in the control group
500 persons treated with both MET and lifestyle intervention with the remaining 1,500
1,500 persons treated with MET or lifestyle intervention with the 500 without any treatment
500 persons treated with both MET and lifestyle intervention with the 500 without any treatment.

In ancillary companion studies we will analyse the epigenetic pattern at baseline, and its change over one year of treatments, in participants who developed, or did not develop, ArCD, as well as in participants who succeeded, or did not succeed, to make MetS regress (with or without MET or lifestyle changes).

Power computations have been based
a) on expected mortality and ArCD incidence in the study area in men and women aged 55 or more (assuming the same age distribution as the general population),
b) on the assumption that MetS is associated with 1,5 higher incidence of ArCD (3 times more for diabetes);
c) on the hypothesis that the planned interventions may reduce ArCD incidence by 25 or 33%; d) on a 3.5-year average follow-up (within the 5-year study period); and,
e) alpha error = 0.05.
Hypothesising to recruit 1000 persons with MS per year in the first two years, excluding the person-years accumulated in the recruitment year, plus 200 persons to compensate the power loss due to expected attrition (for mortality and decisions to stop participation), we would expect about 174 deaths, 175 cancers, and 119 major cardiovascular events (myocardial infarction and stroke), and 219 cases of diabetes, summing up to over 500 incident cases of major ArCD.
We will have, for 33% (*and, respectively, 25%*) reduction in the cumulative incidence of major ArCD (cardiovascular + diabetes + cancer):

- 99% (*92%*) power to compare 1,000 persons treated with metformin with 1,000 receiving placebo (or 1,000 persons with or without active lifestyle intervention)
- 97% (*84%*) power to compare 500 persons treated with both metformin and lifestyle intervention with respect to the other 1,500.
- 89% (*65%*) power to compare 500 persons with both interventions with 500 with none

Comparing 1,000 treated and 1,000 non-treated persons, we will have 69% power to detect a significant 33% difference in total mortality, or in the cumulative incidence of cancer, and 98% power to detect a 33% difference in the cumulative incidence of diabetes (85% for 25% reduction).

c. Resources

The Fondazione IRCCS Istituto Nazionale dei Tumori is fully equipped with infrastructures for gymnasium, dedicated kitchen and cafeteria, teaching rooms, blood collection and sample storage, laboratories for biochemical assays. As the Host Institution has not sufficient qualified personnel for kitchen activities, theses activities as well as the purchase and stocking of food will be assigned to a subcontractor, an agency with knowledge of Mediterranean and Macrobiotic diet and whose tasks will be to buy and prepare meals according to the instructions of the research team, and to teach kitchen technicalities.

Team: The following table describes the size, nature and function of the t

Name	Title	devoted time	Function
Berrino F	MD, pathologist, epidemiologist	40%	PI, study planning & monitoring, life-style conferences and counselling
Pierotti MA	*ScD, PhD, Scientific Director*	10%	Supervisor, consultant for the companion study on the epigenetic effects of metformin and calorie restriction
Pasanisi P	MD, epidemiologist, nutritionist	66%	Planning, medical follow-up, statistical analysis
Villarini A	ScD, biologist, nutritionist	66%	Planning, study organization, quality control, data-base management
Krogh V	MD, epidemiologist	20%	Study design and statistical analysis
Pala V	ScD, agronomist	20%	Dietary & physical activity analysis
Di Mauro G	MD, clinician	50%	Clinical follow-up
Bellegotti M	Dietician	66%	Food diary management, data entry
Bruno E	ScD, nutritionist	80%	Dietary counselling , diary management, data entry
Gargano G	ScD, nutritionist	80%	Dietary counselling , diary management, data entry
Raimondi M	Psychologist	50%	Psychological support, Physical activity trainer
Venturelli E	ScD, biologist	33%	Biological bank, Laboratory determinations
Cavalleri A	Laboratory technician	33%	Biological bank, Laboratory determinations
DelSette D	Secretary, technician	100%	Recruitment, data entry, telephone follow-up
Curtosi P	Secretary	90%	Recruitment, Public relation
Guerrini M	Secretary	20%	Secretariat
Maule A	Cook	50%	Metabolic chef, kitchen classes
Angarano A	Cook	50%	Kitchen classes, preparation of healthy meals

The key team members (Berrino, Pasanisi, Villarini, Venturelli, Allegro, Maule, Angarano) have long experience in managing dietary intervention studies. Names written in italics do not require financial support because they are paid by the host institution. Dr Berrino, former director of the Department of Predictive and Preventive Medicine of the Institute retired and is presently a consultant.

Operative protocol

First meeting
Illustration of the study design, check of inclusion criteria, registration of potentially eligible volunteers, informed consent signature, and arrangement of meeting dates for clinical visit and blood sample

Inclusion criteria:

1. waist circumference > 85 cm for women and >100 cm for men
2. age between 55 and 74
3. no diagnosis of cancer in the last 5 years (except skin carcinomas)
4. no diabetes (IDDM and NIDDM),
5. no kidney insufficiency
6. no acute or chronic hepatitis
7. no diagnosis of coronary heart disease, miocardial infarction, stroke, hearth failure
8. no neurodegenerative disease
9. not under treatment with the following drugs:
   - Cimetidine, Ranitidine
   - Sulfametoxazole + Trimetoprim (Bactrim, Chemitrim, Eusaprim)
   - Nifedipine (Adalat, Nifedicor, Nifesal, Nipin, Fenidina, Euxat, Coral, Citilat)
   - Furosemide (Lasix)
   - Diuretics with K retention: Amiloride (Moduretic), Triamterene, Spironolactone (Aldactone, Uractone, Spirolang)
10. availability to partecipate in the study for 5 years
11. for women aged 55-69, negative mammography in the last 12 months

Second meeting
Clinical visit (including breast examination for women), anthropometric measurements, collection of blood and urine samples

Third meeting
Final eligibility check on the basis of blood and urine examinations

Further inclusion criteria:

1) fasting glycemia <126 mg/dl
2) serum creatinin <124 μmol/L
3) Proteinuria absent or traces (1+)
4) Presence of metabolic syndrome (waist circumference + two further traits)

At this point eligible participants are invited to start taking metformin 425 mg day (half a pill) for 15 days and 850 mg/day (a single full pill) for the subsequent 15 days

The meeting will include:

- clinical visit
- explanation on how to take the pills:
  - in the first 15 days the participant must take half a pill (425mg) of metformin per day, during a main meal (breakfast, lunch or dinner)
  - in the subsequent 15 days the participant must take a full pill (850mg) of metformin per day, during a main meal (breakfast, lunch or dinner)
  - discussion of the possible side effects and invitation to contact the study physician, available 24h/24, in case of symptoms or questions
  - if the participant miss taking one or more pills, he/she must not increase the dose in the following days, and must not throw away the unused pills, which must remain in the box and rendered to the study centre at the next meeting
- arrangement of the next meeting (after 30 days)
- each participant is given a leaflet with instructions

Fourth meeting
Evaluation of compliance and side effects to complete the eligibil
30 days after the start of metformin treatment, the physician will check the participant's compliance through
a) the counting of residual pills
b) specific questions on side effects and on willingness to continue
Participants having demonstrated an adequate compliance and no or trivial side effects will be randomized

RANDOMIZATION

Fifth meeting:
Participants assume a placebo or metformin (850 mg) pill per day for 30 days.
The physician will:
- Provide the pills for the next 30 days
- Remember not throwing away the unused pills
- Arrange the next meeting (that will be also remembered trough an SMS)
- Arrange the dates of kitchen courses for participants randomized in the active lifestyle intervention group

Sixth meeting:
Participants start taking metformin or placebo pills twice a day for one month
The physician will
- Collect information on side effects.
- Count the non used pills to verify compliance.
- Provide the pills for the next 6 months. Participants shall assume two pills per day (for a total of 1700 mg of metformin in the intervention arm); it is recommended to take the pill during the main meals (e.g. one at breakfast and one at dinner, or one at lunch and one at dinner)
- Remember not to increase the dose and not throw away the unused pills
- Stop taking the pills for a few days in case of surgical interventions, anaesthesia, or radiological examinations requiring contrast means
- Arrange the next meeting
- Arrange the dates of common lunches or dinners and for physical exercise meetings for participants randomized in the active lifestyle intervention group

7th and 8th meeting:
Participants, in absence of major side effects, receives the pills for the next 6 months. If the full dose is not tolerated participants are invited to go back to a single pill per day.
9th meeting:
After 12 months of taking metformin or placebo at full dose, participants are invited for a new clinical visit, eligibility check, anthropometric measurements and blood and urine sample.
In case of contra-indication to continue metformin treatment (e.g. for intervening diagnosis of renal insufficiency or major cardiovascular, metabolic or respiratory diseases) participants will be recommended to interrupt the drugs and to continue only the dietary intervention. The participant's general practitioner will be informed.If there are no contra-indications the participants will be given the pills for the next 12 months. At every meeting participants are invited to fill in a food frequency and physical activity questionnaire; this will allow the study dietician to reinforce lifestyle recommendations for those randomized in the active lifestyle intervention group.Participants randomized in the intervention group not showing significant improvements will be offered special teaching sessions.

Further yearly meeting:
To check eligibility, body weight and waist circumference, and to provide the pills for the next 12 months
Every meeting will be preceded by a telephone remind (SMS)
At each meeting and in a random day every two months all participants will be invited (through SMS or telephone) to fill in a 24h food frequency and physical activity diary
For participants randomized into the dietary intervention group the meetings will usually arranged in the same days of the kitchen courses and common meals.
Participants who for whatever reason decide to interrupt the study will be invited for a final clinical visit and anthropometric measurement

c. Funding

Amount of funding necessary
The estimates are largely based on what we spent for our ongoing life-style intervention study of 2,000 breast cancer survivors.

Drug
On average participants will be treated for 4 years with either Metformin or placebo. Our pharmacist has estimated that we will need 400,000 Euro to prepare Metformin and placebo for the whole study. Metformin is very cheap (less than 5 Euro per month of treatment) but companies are not interested to sponsor a study.

Food and kitchen activities
On average participants randomized in the intervention group will receive 4 full-day kitchen courses and will be invited monthly for lunch or dinner with short kitchen courses for 4 years (10 months per year), for a total of 40,000 meals. In previous trials we spent 7 Euro per meal. Taking into account inflation but also some scale economy the total expenditure for food and kitchen activities will be 8 x 40,000 = 320,000 Euro (125,000 about for food purchase).

Laboratory activities
Laboratory costs are mainly for basic clinical chemistry and insulin, growth factors, and inflammatory cytokines. For epigenetic tests we have applied to other sources.

**Equipment**	
-80°C Freezer	Euro 20,000
**Consumables**	
Metformin	400,000
Vials for biological bank (16 per person)	10,000
Baseline and 12 month laboratory exams (20 Euro x person)	40,000
Hormonal exams	45,000
**Subtotal Consumables**	495,000
**Travels** (participation to conferences/congress to present data and local trips to organize recruitment)	10,000
**Publication** costs (journal fees)	5,108
**Personnel (see attached form)**	1,340,500
**Indirect costs (20%)**	374,121.60
Subcontract for kitchen activities and food purchase	234,000
Certificates of Financial Statement (CFS)	21,270
**Subtotal subcontracting costs:**	255,270
**Total**	**2,499,999,60**

	Cost Category	Month 1-18	Month 19-36	Month 37-54	Month 55-60	Total (M1-60)
**Direct Costs:**	*Personnel:*					
	PI[1]	20,000.00	60,000.00	60,000.00	20,000.00	160,000.00
	Senior Staff	260,000.00	260,000.00	260,000.00	120,000.00	900,000.00
	Post docs	57,500.00	57,500.00	57,500.00	22,000.00	194,500.00
	Students					
	Other	20,000.00	25,000.00	25,000.00	16,000.00	86,000.00
	Total Personnel:	357,500.00	402,500.00	402,500.00	178,000.00	1,340,500.00
	Other Direct Costs:					
	Equipment	6,000.00	6,000.00	6,000.00	2,000.00	20,000.00
	Consumables	125,000.00	160,000.00	160,000.00	50,000.00	495,000.00
	Travel	2,500.00	2,500.00	2,500.00	2,500.00	10,000.00
	Publications, etc				5,108.00	5,108,00
	Total Other Direct Costs:	133,500.00	168,500.00	168,500.00	59,608.00	530,108.00
	Total Direct Costs:	491,000.00	571,000.00	571,000.00	237,608,00	1,870,608.00
**Indirect Costs (overheads):**	Max 20% of Direct Costs	98,200.00	114,200.00	114,200.00	47,521.60	374,121.60
**Subcontracting Costs:**	Audit	6,700.00	7,350.00	7,220.00		21,270.00
	Kitchen activities and food purchase	70,000.00	70,000.00	70,000.00	24,000.00	234,000.00
**Total Subcontracting Costs:**		76,700.00	77,350.00	77,220.00	24,000.00	255,270.00
**Total Costs of project:**	(by reporting period and total)	665,900.00	762,550.00	762,420.00	309,129.60	2,499,999.60
**Requested Grant[2]:**	(by reporting period and total)	665,900.00	762,550.00	762,420.00	309,129.60	2,499,999.60

Time table and milestones

1-18 Month: Formalisation of the operative protocol, Start of recruitment
19-36 Month: Recruitment, Start of follow-up, Quality control checks, Second blood sample
37-54 Month: Follow-up, Hormonal and cytokines exams, Ad-interim analyses
55-60 Month: End of follow-up, Statistical analysis

**For the above cost table, please indicate the % of working time the PI dedicates to the project over the period of the grant:**	**40%**

Dr Franco Berrino "PI" will follow all the phases of the project. He will be personally involved in the counselling of participants and in the quality control of procedures and outcomes.

iii. Budget - Table 2

„key intermediate goal", as defined in section 2.	Estimated % of total requested grant	Expected to be completed on month :	Comment
formalization of the operative protocol, start recruitment	28%	18	In this fist period we have to implement and test strategies for efficient recruitment
complete recruitment, start follow-up, compliance monitoring	31%	36	Active follow-up will start as soon as the first recruited persons reach one year of treatment
follow-up, biochemical analyses	30%	54	Metabolic and hormonal analyses will be used to proceed to a first comparison between the four arms of the study
end follow-up, quality control of end-points, statistical analysis	11%	60	During all the study any effort will be made to obtain clinical and pathological reports of the diagnosed diseases. The main analyses will be by intention to treat.
**Total**	**100%**		

d. Ethical and security-sensitive issues

The Study has been submitted for approval to the Institutional Review Board and Ethical Committee of the Host Institution.

ETHICS ISSUES TABLE

	Research on Human Embryo/ Foetus	YES	Page
	Does the proposed research involve human Embryos?		
	Does the proposed research involve human Foetal Tissues/ Cells?		
	Does the proposed research involve human Embryonic Stem Cells (hESCs)?		
	Does the proposed research on human Embryonic Stem Cells involve cells in culture?		
	Does the proposed research on Human Embryonic Stem Cells involve the derivation of cells from Embryos?		
	I CONFIRM THAT NONE OF THE ABOVE ISSUES APPLY TO MY PROPOSAL	X	

	Research on Humans	YES	Page
	Does the proposed research involve children?		
	Does the proposed research involve patients?		
	Does the proposed research involve persons not able to give consent?		
	Does the proposed research involve adult healthy volunteers?	X	6, 9-12
	Does the proposed research involve Human genetic material?	X	9, 10
	Does the proposed research involve Human biological samples?	X	9, 10
	Does the proposed research involve Human data collection?	X	9,10,12
	I CONFIRM THAT NONE OF THE ABOVE ISSUES APPLY TO MY PROPOSAL		

	Privacy	YES	Page
	Does the proposed research involve processing of genetic information or personal data (e.g. health, sexual lifestyle, ethnicity, political opinion, religious or philosophical conviction)?	X	9,10
	Does the proposed research involve tracking the location or observation of people?	X	9,12
	I CONFIRM THAT NONE OF THE ABOVE ISSUES APPLY TO MY PROPOSAL		

	Research on Animals[3]	YES	Page
	Does the proposed research involve research on animals?		
	Are those animals transgenic small laboratory animals?		
	Are those animals transgenic farm animals?		
	Are those animals non-human primates?		
	Are those animals cloned farm animals?		
	I CONFIRM THAT NONE OF THE ABOVE ISSUES APPLY TO MY PROPOSAL	X	

[3] The type of animals involved in the research that fall under the scope of the Commission's Ethical Scrutiny procedures are defined in the Council Directive 86/609/EEC of 24 November 1986 on the approximation of laws, regulations and administrative provisions of the Member States regarding the protection of animals used for experimental and other scientific purposes Official Journal L 358 , 18/12/1986 p. 0001 - 0028

	Research Involving non-EU Countries (ICPC Countries[4]) [5]	YES	Page
	Is the proposed research (or parts of it) going to take place in one or more of the ICPC Countries?		
	Is any material used in the research (e.g. personal data, animal and/or human tissue samples, genetic material, live animals, etc) : a) Collected in any of the ICPC countries?		
	b) Exported to any other country (including ICPC and EU Member States)?		
	I CONFIRM THAT NONE OF THE ABOVE ISSUES APPLY TO MY PROPOSAL	X	

	Dual Use	YES	Page
	Research having direct military use		
	Research having the potential for terrorist abuse		
	I CONFIRM THAT NONE OF THE ABOVE ISSUES APPLY TO MY PROPOSAL	X	

[4] In accordance with Article 12(1) of the Rules for Participation in FP7, 'International Cooperation Partner Country (ICPC) means a third country which the Commission classifies as a low-income (L), lower-middle-income (LM) or upper-middle-income (UM) country. Countries associated to the Seventh EC Framework Programme do not qualify as ICP Countries and therefore do not appear in this list.

[5] A guidance note on how to deal with ethical issues arising out of the involvement of non-EU countries is available at:
ftp://ftp.cordis.europa.eu/pub/fp7/docs/developing-countries_en.pdf
